# Current Trends of Microfluidic Single-Cell Technologies

**DOI:** 10.3390/ijms19103143

**Published:** 2018-10-12

**Authors:** Pallavi Shinde, Loganathan Mohan, Amogh Kumar, Koyel Dey, Anjali Maddi, Alexander N. Patananan, Fan-Gang Tseng, Hwan-You Chang, Moeto Nagai, Tuhin Subhra Santra

**Affiliations:** 1 Department of Engineering Design, Indian Institute of Technology Madras, Tamil Nadu 600036, India; shindepallavi25@gmail.com (P.S.); mohanbioengineer@gmail.com (L.M.); amogh.kb@gmail.com (A.K.); koyeldeykoyeldey1992@gmail.com (K.D.); maddianjali@gmail.com (A.M.); 2 Department of Pathology and Laboratory Medicine, University of California, Los Angeles, CA 90095, USA; apatanan@g.ucla.edu; 3 Department of Engineering and System Science, National Tsing Hua University, Hsinchu City 30071, Taiwan; fangang@ess.nthu.edu.tw; 4 Department of Medical Science, National Tsing Hua University, Hsinchu City 30071, Taiwan; hychang@life.nthu.edu.tw; 5 Department of Mechanical Engineering, Toyohashi University of Technology, Toyohashi 441-8580, Japan; nagai@me.tut.ac.jp

**Keywords:** single-cell, lab-on-a-chip, microfluidics Bio-MEMS, manipulation, diagnosis, therapeutics, drug delivery

## Abstract

The investigation of human disease mechanisms is difficult due to the heterogeneity in gene expression and the physiological state of cells in a given population. In comparison to bulk cell measurements, single-cell measurement technologies can provide a better understanding of the interactions among molecules, organelles, cells, and the microenvironment, which can aid in the development of therapeutics and diagnostic tools. In recent years, single-cell technologies have become increasingly robust and accessible, although limitations exist. In this review, we describe the recent advances in single-cell technologies and their applications in single-cell manipulation, diagnosis, and therapeutics development.

## 1. Introduction

Cells are the fundamental operating units in living organisms. In multicellular organisms, cells communicate over small distances through direct contacts or over extended distances through chemical signals regulated by the microenvironment [1,2,3]. Such cell-cell and cell-matrix interactions are based on the gene expression of individual cells, with detrimental genetic aberrations leading to the development of diseases. The treatment of many diseases, such as diabetes, cancer, and neurodegenerative disorders, remains difficult due to the complex interaction of molecules and signaling pathways in and between cells [4,5,6]. To establish more effective treatments for these diseases, it is essential to target biomolecules and their respective cellular pathways to alter pathological conditions [7,8,9].

Over the past two decades, the ability to interrogate molecular and cellular pathways has improved rapidly [10,11,12,13,14] and has contributed to our better understanding of several diseases and the design of useful drugs [15,16,17]. However, as the throughput and complexity of various technologies designed to interrogate biological systems have increased, the cost and time associated with analyzing such large samples have also increased. Furthermore, many approaches to the study of cell biology involve the culturing of many cells together in a heterogeneous physiological state with different gene and biomolecule expression profiles. Data collected in such a bulk culture tend to lose low-frequency information [18,19] that may affect the accuracy of diagnosis and delay therapy [20,21,22]. Thus, it has become apparent that innovative methodologies are required to perform these analyses. The development of lab-on-a-chip, along with bio-micro electro mechanical systems (Bio-MEMS) or micro total analysis system (µTAS), may serve as a methodology that can provide high throughput data, with limited reagent consumption and assay complexity [23,24,25,26]. These systems are designed for single-cell assays at the 10–100 µm scale. Active monitoring of a single-cell in a well-controlled environment without the influences of nearby cells is essential to understand cell physiology.

Single-cell technologies (SCT) are a set of novel techniques developed for studying single-cells and their interactions with other cells and the microenvironment in well-defined conditions [27,28,29]. In contrast to bulk analysis that provides average data for a population, SCT can identify different cellular characteristics and molecular dynamics in a cell population, which is particularly useful in characterizing tumor-initiating cells [28,29].

In order to analyze single-cell characteristics, data can be collected from individual cells in static or dynamic conditions so that phenotypic and functional alterations in diseased cells can be traced back to genetic aberration. Bioinformatics techniques can be used to handle the collection of such large amounts of diverse data generated from individual cells [27]. In the last two decades, the rapid development of Lab on a Chip has demonstrated that single cell analysis is a powerful technique for cell manipulation, diagnostics, and therapeutic developments [27,30,31].

Here, we demonstrate different physical approaches for single cell manipulation such as dielectrophoresis, optical, acoustic, and microfluidic microprobes. Furthermore, single cell separation and diagnosis techniques are discussed using microfluidic Bio-MEMS devices integrated with photomechanical, laser capture micro dissection, and nano-capillary chip technologies. Single cell therapeutic and analysis techniques are performed using electroporation [32], optoporation, mechanoporation [33], capillary electrophoresis coupled with laser-induced fluorescence detection, flow cytometry, mass spectrometry, high-resolution microscopy, the resonating microelectromechanical system (MEMS), electro neural nanowire device, and nanostraw system. Thus, this review article intends to provide an overview of currently available techniques for single cell manipulation, analysis, and their applications in diagnostic as well as therapeutic development. The research perspective, future prospects of SCT, and its applications will also be discussed.

## 2. Single-Cell Manipulation

In-vitro cell growth conditions are often insufficient in reconstituting the cellular microenvironment at the high spatial resolution and cannot eliminate the interference of chemicals released from adjacent cells. Thus, establishing an ability to place a single-cell in the desired location through “*single-cell manipulation*” is essential for the investigation of cell physiology.

A range of procedures for the physical displacement of single-cells has been developed. These procedures include fluidic-based [34,35,36,37], physical-based [38,39,40,41], electric field driven approaches such as dielectrophoresis (DEP), optoelectronic tweezers (OET) [42,43,44,45,46,47,48], and optical techniques such as optical tweezers [49,50,51,52,53,54]. The basic theories, advantages, and disadvantages of these technologies are briefly described below (Table 1). Also discussed are recent developments in parallel single-cell manipulation.

### 2.1. Fluidic-Based Manipulation

#### 2.1.1. Droplet Microfluidics

Droplet microfluidics involves compartmentalizing reactions by isolating molecules, particles or cells into droplets [55]. This is achieved by isolating the target particles in aqueous microdroplets, which are separated by immiscible oil or similar fluids. This two-phase system allows for a level of control on the picoliter scale. Droplet-based microfluidic devices are valuable tools for single-cell handling due to their capability of isolating cells for further analysis. These cells can be transported through microchannels for a variety of downstream applications. A lab on a chip for digitally controlling single cell encapsulation and independent aqueous reagent microdroplet reactors for screening cell viability and cytotoxicity has been proposed by Brouzes et al. [75]. Allowing higher flexibility and feasibility to handle microdroplets, the aqueous medium is replaced by hydrogels as discussed by Zhu et al. [76]. A comprehensive overview of droplet microfluidics has been discussed by Shang et al. [56].

**Figure 1:**
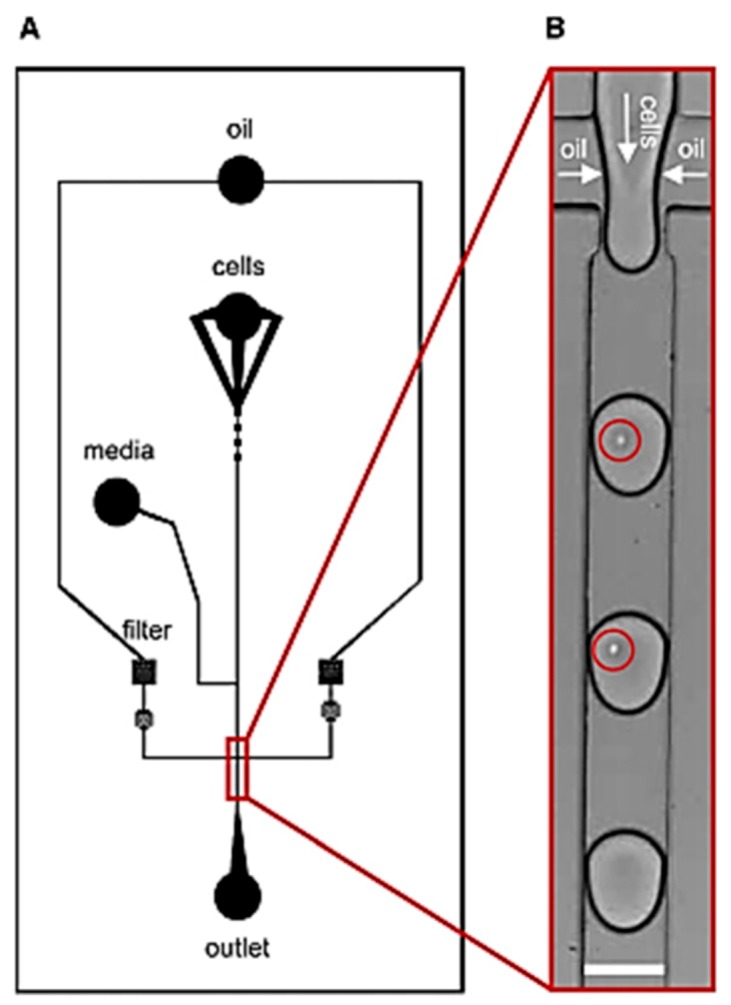
Schematic diagram of the chip proposed by Jenifer et al. (**A**) The bounded rectangle shows the area where droplets containing cells are generated; (**B**) Mechanism of encapsulation of the cells in the aqueous droplets. The red circles highlight the single cells in the droplets. Reprinted with permission from [57].

Jenifer et al. [57] showed the application of droplet-based microfluidic platforms in high throughput screening of Human Embryonic Kidney cells (HEK293T) (Figure 1). The microdroplets were generated by flow focusing a stream of cell suspension diluted in cell media, with perfluorinated oil. The concentration of nutrients inside the droplets was altered by varying the relative flow rates of the cell media and the cell suspension. The proliferation of both adherent and non-adherent cells in these microdroplets was observed for several days. Even after 14 days of incubation, greater than 90% of the drops remained intact, showing a very low degree of coalescence.

**Figure 2:**
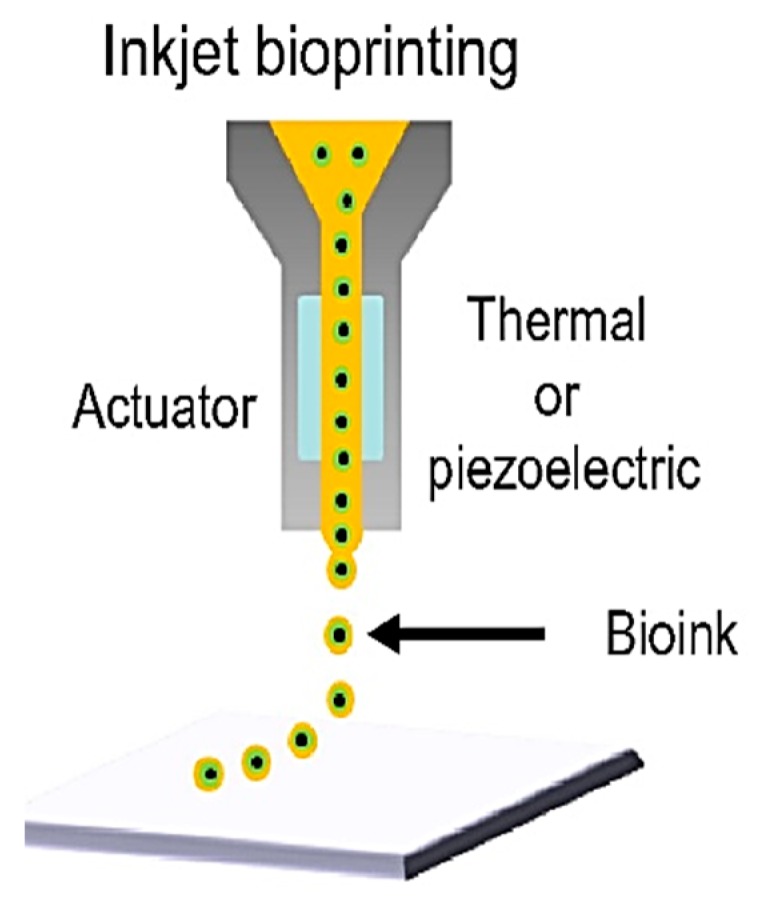
Schematic representation of Inkjet printers ejecting small droplets of cells and hydrogel sequentially to build up tissues. Reprinted with permission from [77].

While the above techniques introduce devices where isolated droplets are generated on-chip, Yusof et al. reported a device for dispensing droplets into other devices via an inkjet-like single-cell printing technology by means of a piezo-actuator [69]. The schematic representation of inkjet printers is shown in Figure 2 [77]. While these devices are adept in terms of highly flexible cell manipulation, they are limited in their output because of the use of a single probe. Maintaining flexibility, accessibility, and increasing the number of probes favors higher throughput in single-cell manipulation. The single probe-tip device is appropriate for flexible cell manipulation.

#### 2.1.2. Microfluidic Deterministic Lateral Displacement Technology

**Figure 3:**
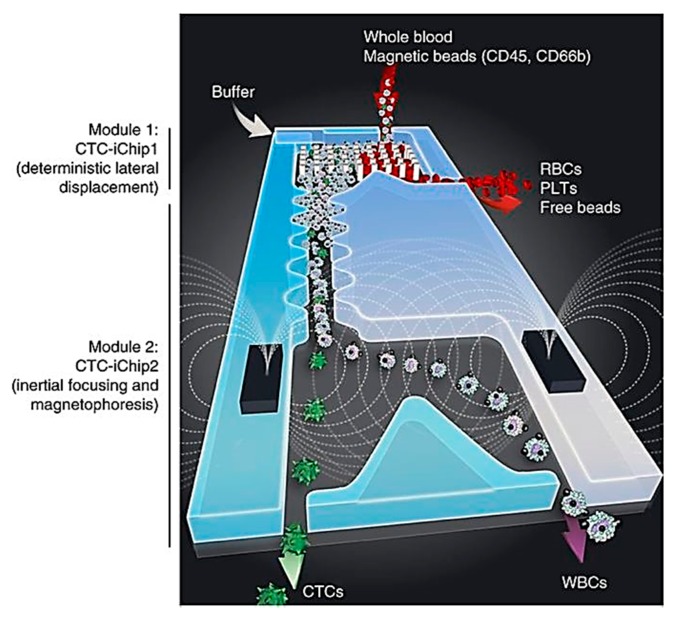
Schematic representation of CTC iChip showing two stages: (1) iChip1 uses the LDL technique for separating red blood corpuscles (RBCs), platelets (PLTs) and free beads from the whole blood; (2) iChip2 uses magnetophoresis for separating CTC from white blood cells (WBCs). Reprinted with permission from [59]. Copyright (2014) Springer Nature.

Using microstructures to control the fluidic field is a convenient way to isolate single cells. The major advantage of using fluidic-based cell manipulation is its simplicity. No exogenous electric or magnetic or optical fields need to be applied, which can complicate the instrument design and result in unwanted effects on cells. Numerous microfluidic chips have used microfilters that prevent cells of certain sizes from passing through [78,79]. More complex microstructures such as micropillar arrays can be used to separate cells of different sizes. One such design facilitated the isolation of circulating tumor cells from blood samples [80]. The CTC-iChip developed by Karabacak et al. [59] provides a novel way for the isolation of CTCs by using a two-stage magnetophoresis process (Figure 3). In the first stage, a continuous-flow system using deterministic lateral displacement (DLD) is used to separate the nucleated cells from blood. This is done by specifying a critical deflection diameter (D_c_) for the DLD pillar arrays, such that the particles of interest have a diameter smaller than D_c_ and pass through the DLD pillar array without being deflected; while the particles having diameters larger than D_c_ would get deflected by the array. The second stage involves the use of inertial focusing to enable the precise lateral positioning of particles in a microfluidic channel, which then allows for highly efficient separation of target cells by magnetophoresis.

Kirby et al. modified DLD to develop a new microfluidic device called geometrically enhanced differential immunocapture (GEDI). This device has a pillar array designed to increase targeted cell to pillar collisions during the flow path. This is achieved by displacing the pillar rows with an incremental offset. These pillars are functionalized with the targeted cell’s antigen recognizing antibody. This results in the capture of targeted cells and undesired cell interactions are avoided. This technique has been successfully used for prostrate, breast, and gastric CTCs with a 2–400-fold increase in sensitivity [60].

**Figure 4:**
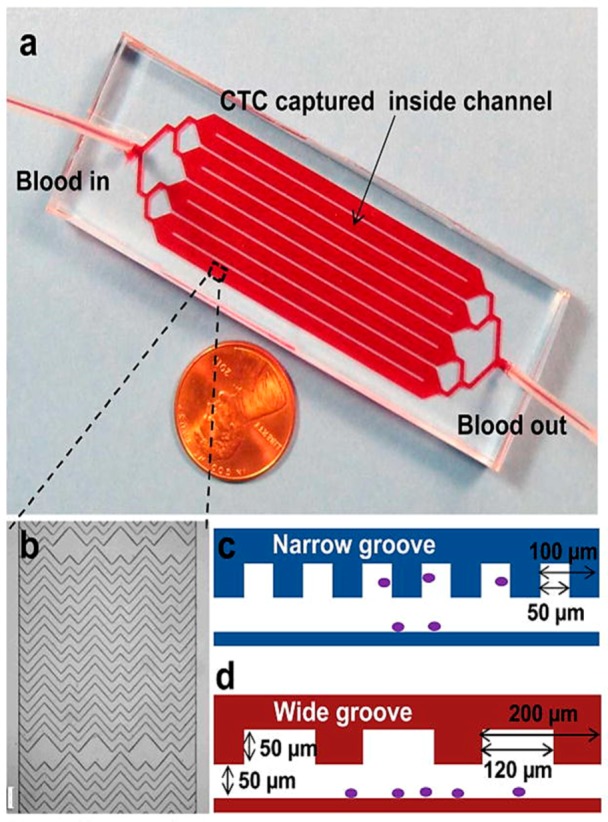
(**a**) Picture of a Microfluidic GEM chip; (**b**) Micrograph of the channel showing grooves inside the channel useful in cell capture; (**c**) Narrow groove (with dimensions) showing captured cells (purple); (**d**) Cross sectional view showing the wide groove (with dimensions). “Reproduced from [61] with permission of The Royal Society of Chemistry”.

Another antibody-based CTC cell trapping technique, geometrical enhanced mixing (GEM), has been developed by Sheng et al. [61] (Figure 4). It consists of a microfluidic mixer with optimized geometry for increasing transverse flow and flow folding to increase the cell interaction with antibody functionalized surfaces. The device achieves up to 90% cell capture efficiency with 84% purity.

#### 2.1.3. Hydrodynamic Pressure Based Fluidic Manipulation

**Figure 5:**
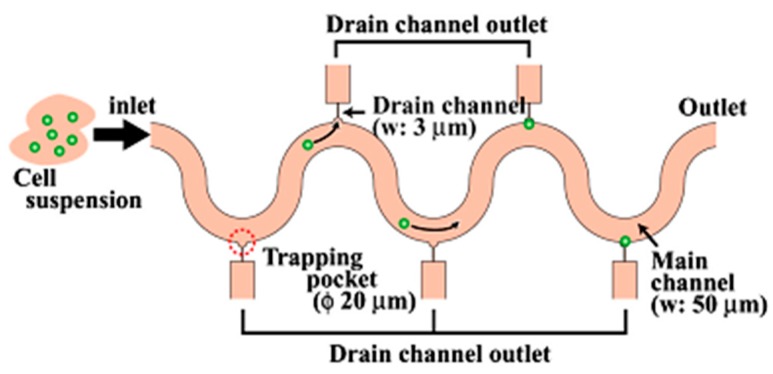
Trapping of single cells using the device designed by Arakawa et al. The cells can either be caught by the drain channels or continue flowing in the main channel as denoted by the arrows. “Reproduced from [62], with the permission of AIP Publishing”.

T Arakawa et al. [62] introduced a device that captures cells in small trapping micropockets, enabling further single cell processes such as isolation, tagging, and single-cell rupture (Figure 5). The device consists of microchannels that are serpentine in shape and consist of 64 or 512 trapping sites in each channel in series. The device allows cell trapping followed by isolation, tagging and rupture in succession in the same channel the cells are captured in. The serpentine channels’ geometric parameters are optimized based on cell size using finite element fluidic analysis. Arakawa et al. observed a capture rate of 70–83.2% in their experiments. The semicircular trapping pockets dimensions are decided as per the size of cells considered, to prevent the cells from washing away due to the sudden flow in the main channel. Finally, cell rupture is performed by injecting surfactant into the main channel. The cell membranes rupture and the cells’ contents are obtained by applying a negative pressure through the collection channels. Thus, cell trapping, tagging and rupture can be performed in succession on the same device.

**Figure 6:**
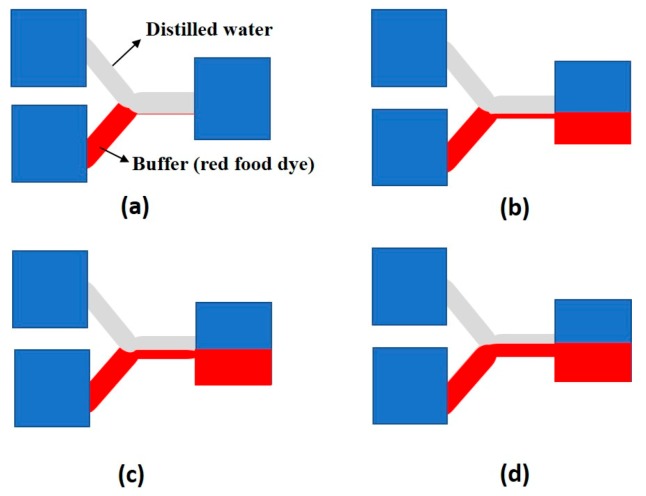
Schematic of different levels of the sample (distilled water) and the buffer (red food dye) in the common channel as per the ratio of the sample input pressure P_s_ and the buffer input pressure P_b_ in the device designed by Bragheri and Osellame. (**a**) (P_s_/P_b_) = 1.85. (**b**) (P_s_/P_b_) = 1.6. (**c**) (P_s_/P_b_) = 1.2. (**d**) (P_s_/P_b_) = 1.85. Redrawn from [63].

Bragheri and Osellame [63] introduced a chip to enable single cell manipulation by varying pressures from two sources: an input sample containing the cell suspension, and a buffer sample (Figure 6). The ratio of the pressures of input flowing from these two sources is the major factor determining the level of the buffer layer in the common channel. Both the sources originate from different heights and since the flows are laminar, the levels of both the samples can be controlled by just altering their input pressures. Lincoln et al. [64] showed that cells or microbeads tend to flow at the bottom of a channel under low flow rates. Thus, the positions of the cells in the common channel can be manipulated using this device.

#### 2.1.4. Fluidic Microarray Chip for Cellular Manipulation

One of the problems faced when dealing with cells populations is that they typically are present in huge numbers and are individually vulnerable. Processing a large number of cells renders sophisticated experiments lengthy and difficult to repeat when one cell is handled at a time. In addition, one doesn’t want to lose important information from SCT during such procedures. Hence, it becomes essential to manipulate the maximum possible number of cells simultaneously while being consistent in maintaining their individuality. Several researchers have established the possibility of developing 2-D microwell arrays on semiconductor substrates or micromachining processes to achieve a highly parallel assay on a microchip [81,82,83].

**Figure 7:**
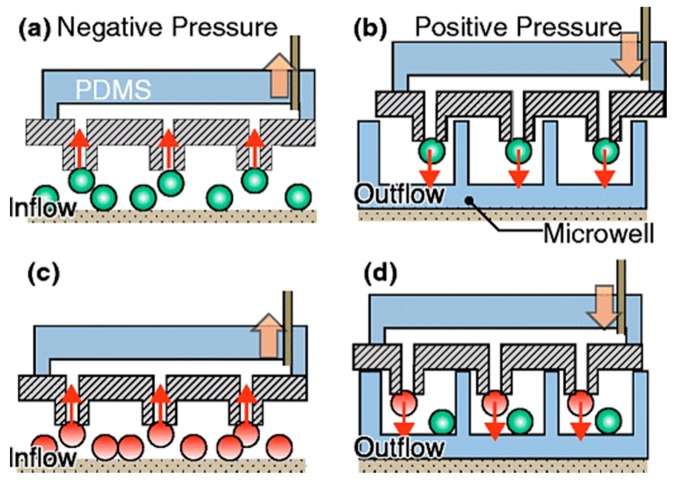
Schematic representation of parallel manipulation of single-cells: The orange arrows represent the direction of movement of air on application of pressure and the red arrows represent the direction of the movement of cells due to applied pressure. (**a**) Pattern-based trapping of one type of single-cells; (**b**) Releasing the cells after transportation; (**c**) Suction of another type of single-cells; (**d**) Transport of another type of cells for co-culture. Reprinted with permission from [70]. Copyright (2015) Springer Nature.

Holding a cell through a glass pipette by suction flow is a standard technique for single-cell manipulation [84]. However, a recent parallel manipulation of single-cells by forming an array was reported by Nagai et al. [70], who described a device that comprised of 100 microprobes within a chip for single-cell manipulation. Figure 7 shows the principle of this array-based platform, where each microprobe in the array acted as a micropipette. A suction force is created by holding the chip over the desired cell culture matrix and applying negative pressure to the chip through a polydimethylsiloxane (PDMS) pump.

The dimension of the probe aperture and the suction pressure is optimized in such a way that individual cells are held by each probe. A microwell array is also designed such that individual cells can be released in each well on applying positive pressure through the PDMS pump to the chip. This study may promote a mechanical model for minimally invasive single-cell manipulation.

Hong et al. [36] and Chen et al. [37] effectively executed a cell-cell communication platform that can be used to co-culture a pair of cells in one chamber. For effective single-cell pairing, Frimat et al. [35] established a new cellular valving model to use within a differential resistance microfluidic circuit. Microfluidic control of cell pairing and fusion was studied by Skelley et al. [34]. Cell fusion was attained in an array format once the cells were paired [36,37]. The flexibility of the design for the use of different cell numbers and cell types can be improved using these chips.

**Figure 8:**
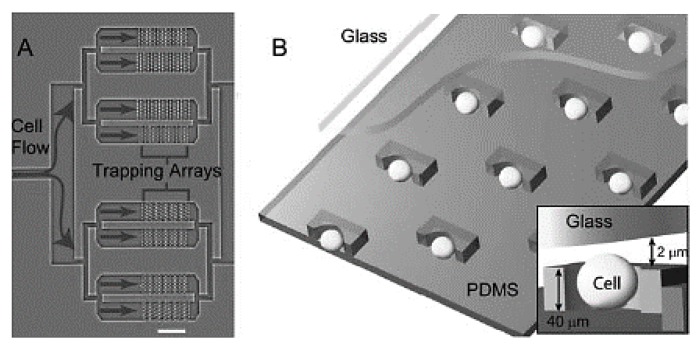
(**A**). Architecture of the system proposed by Di Carlo et al. The scale bar is 500 µm; (**B**). Diagram of the microfluidic chip and the trapping mechanism. “Reproduced from [65] with permission of The Royal Society of Chemistry”.

Di Carlo et al. [65] proposed a device for microfluidic-based culture of cells in ordered arrays with controlled dynamic perfusion; shown in Figure 8. They identified important parameters for the device, such as higher trapping efficiency observed in case of asymmetry in offset distances of rows as compared to symmetrically offset rows. They also observed the ideal depth to vary with the average size of the cell. In their experiments with HeLa cells, they found the single cell initial trapping rate to be 70% with the proposed device and above 85% retention of the cells in the trapped sites. The cells were shown to be adherent to the PDMS trapping sites and only 5% of the cells were apoptotic after 24 h, while 1% had undergone cell division.

**Figure 9:**
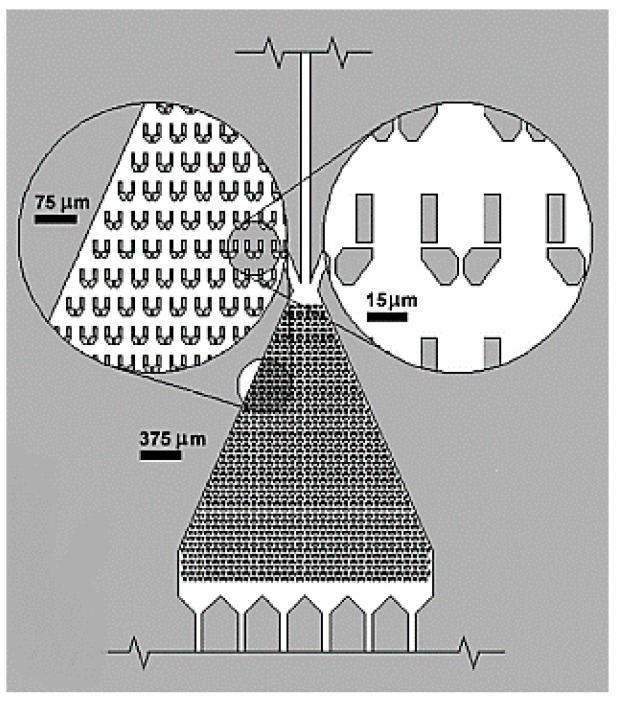
CAD schematic representation of the microfluidic device proposed by Wlodkowic et al. showing the traps in the triangular array. Reprinted with permission from [66]. Copyright (2009) American Chemical Society.

Wlodkowic et al. [66] introduced a microfluidic platform for screening of anticancer drugs against single cells trapped in arrays. The device consists of an array of traps fabricated with PDMS (Figure 9). It showed a trapping efficiency of 10–20% for both U937 and HL60 cells in their experiments and over 85% of the cells initially trapped retained their position even after cell perfusion. Wlodkowic et al. also discussed the potential of the device in proliferation of the cells after trapping [67]. Thus, the microfluidic device allows for real-time trapping and screening of cells in small populations.

**Figure 10:**
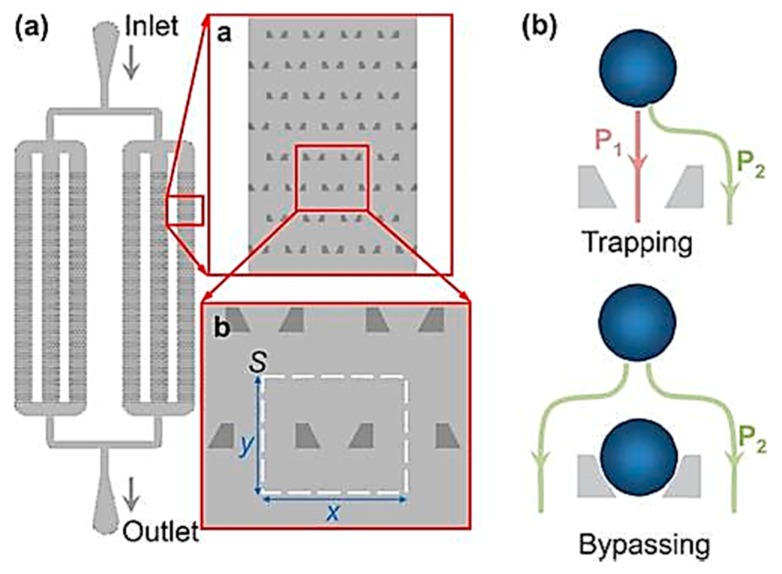
Schematic representation of the microfluidic microsphere-trap array. (**a**) Layout (top view): Microfluidic channels with hydrodynamic trap arrays. The channels are connected by a common inlet and a common outlet. Liquid solution carrying the microspheres flows from the inlet and through the chamber; (**b**) Trapping mechanism. Reproduced from [68] with the permission of AIP Publishing.

Xu et al. [68] proposed a novel cell trapping microfluidics-based device and developed a robust framework to identify and optimize parameters to maximize trapping efficiency in the proposed device (Figure 10). The device first captures microspheres flown into it via the PDMS-fabricated traps followed by capture of target cells on the trapped microspheres. The microspheres have embedded receptors to capture the cells [85]. The optimized device reported a trapping efficiency of 99% and a high trap density of 1438 traps/mm^2^ in their experiments.

### 2.2. Electric Field-Driven Manipulation

Manipulation and patterning of cells can be achieved through the application of dielectrophoretic (DEP) conditions using non-uniform electric fields [71,72]. Hunt et al. 2006 & Kodama et al. 2013 reported on dielectrophoretic tweezer techniques as an alternative to suction for cell manipulation [86,87]. Lee et al. utilized the DEP tweezer technology for studying particle surface interaction in microfluidic devices, which further developed into a force spectroscopy technique [88]. Kim et al. automated the DEP tweezer-based force spectroscopy technique to manipulate particles in 3D [89].

### 2.3. Optical Manipulation

**Figure 11:**
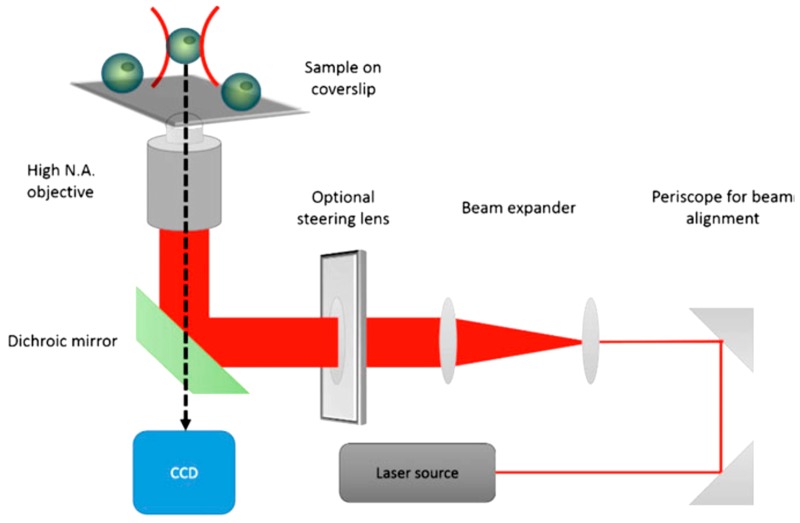
Schematic diagram of optical tweezers used to manipulate single cells. Reprinted with permission from [91]. Copyright (2016) Springer Nature.

Optical tweezers are widely used for single cell optical manipulation. Figure 11 shows the schematic diagram of optical tweezers. When light strikes an object, its scattering is always accompanied with some momentum transfer to the object as per the law of conservation of momentum. This implies that while some photons in light beams get scattered off the incident surface of the object, some impart momentum to it in the direction of the beam. When a dielectric object is irradiated by a Gaussian laser beam, dielectric particles are attracted towards the center of the beam, since it is the region where the electric field is strongest. Thus, any small displacement away from the center of the beam causes a restoring force generated by the electric field gradient to act on the particle, pushing it back towards the center of the beam. In addition, the net pressure from the photons in the direction of the beam applies a positive force on the particle along the direction of beam propagation [90,91].

This technique can be effectively employed in precise single cell handling. The advantages of using optical methods have been reported by Chiou et al. 2005, Mirsaidov et al. 2008, and Juan et al. 2011. However, optical tweezers are prone to light diffraction and impose limitations on available substrates when handling multiple cells at the same time [49,50,51]. Liberale et al. [92] presented a new approach to integrating optical trapping with microfluidic devices for on-chip manipulation and analysis by using miniaturized optical tweezers. Their device shows great potential in cell manipulation and screening due to its versatility and ease of fabrication, whilst also ensuring cell safety.

**Figure 12:**
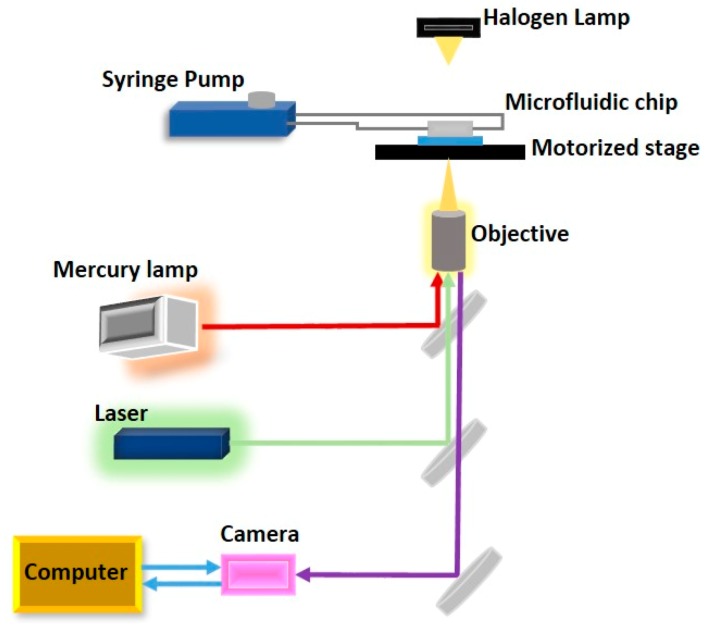
Schematic representation of cell manipulation using computer-controlled motorized stage. Redrawn from [93].

To achieve parallel manipulation of cells, Wang et al. [73] combined hydrodynamics with the holographic optical tweezing operation to create multiple 3D optical traps to separate cells from a mixture (Figure 12). The 3D optical traps are integrated into a microfluidic device with an array of microwells to dock cells that pass over it through a microfluidic channel placed on a motorized stage. The docked cells are then observed through a microscope that scans the array to capture the image. Using an image processing technique, the desired cells are detected and an optical trap is created to trap specific cells using a continuous wave laser source. Multiple traps are created depending on the number of cells detected. The *Z* stage of the objective moves vertically upwards to levitate the trapped cells. The motorized stage then moves in an *X*–*Y* direction to transport the cells to the desired location. The entire process is controlled by software. Wang et al. reported a successful levitation rate of 78.5 ± 5.4% and a successful transfer rate of 97 ± 1.41% using this proposed system [93].

This technique achieves the parallel manipulation of cells. However, it requires a sophisticated experimental setup. In addition, the number of optical traps that can be generated is limited by the maximum laser power.

**Figure 13:**
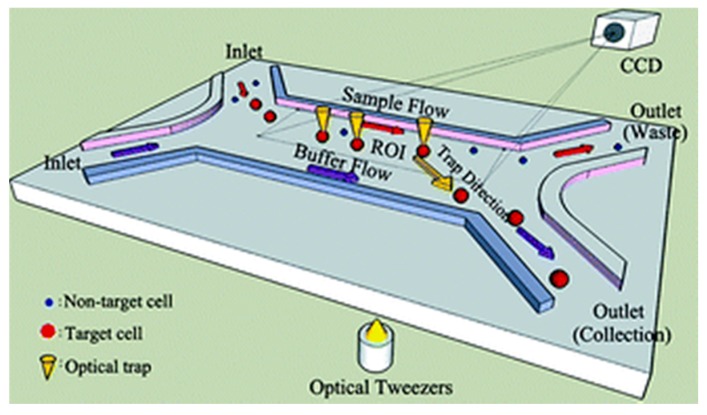
Schematic representation of the cell sorting procedure. Reproduced from [94] with permission of The Royal Society of Chemistry.

Wang et al. [94] introduced a system integrating optical tweezers into microfluidic technology for cell isolation, transport and deposition in a non-invasive manner (Figure 13). Their system uses digital image processing to identify important features such as cell size and fluorescence to identify target cells. The optical traps can be generated by their system at any position inside the region of interest to trap the cells once they are detected by the image processing module. To capture the cells, the fluid drags force, and the optical trapping force must neutralize each other so that the cell moves at a constant velocity and can be moved from the sample flow to the buffer flow using the optical tweezers module. They demonstrated the working of this system using Human Embryonic Stem cells and reported high purity and recovery rate of the target cells from the input sample.

### 2.4. Acoustic Based Mainpulation

**Figure 14:**
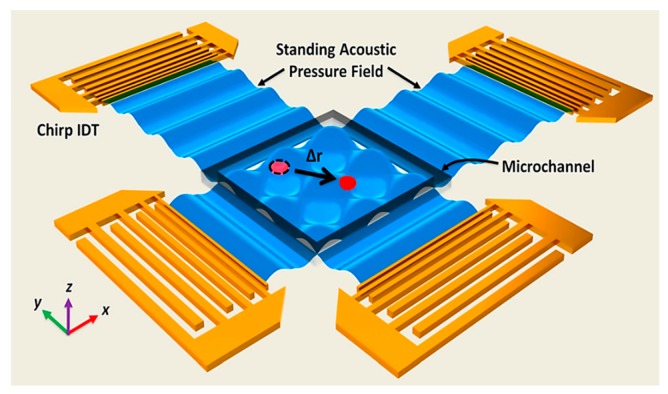
Schematic diagram showing the mechanism of the device proposed by Ding et al. Permission to reprint obtained from PNAS [95].

Ding et al. introduced the first acoustic tweezers (Figure 14), which showed precision close to those of optical tweezers while having a power density orders of magnitude lesser than those of optical tweezers (10,000,000 times lesser) and optoelectronic tweezers (100 times lesser), thus making acoustic tweezers way more biocompatible. The device was employed in 2D acoustic manipulation of HeLa cells and micro-organisms by real-time control of a standing surface acoustic wave field. The device showed the ability of moving cells across the platform at a very high speed of up to 1600 μm/s. They used polystyrene microparticles to show how the device enabled precise and intricate manipulation on the 2D platform [95].

**Figure 15:**
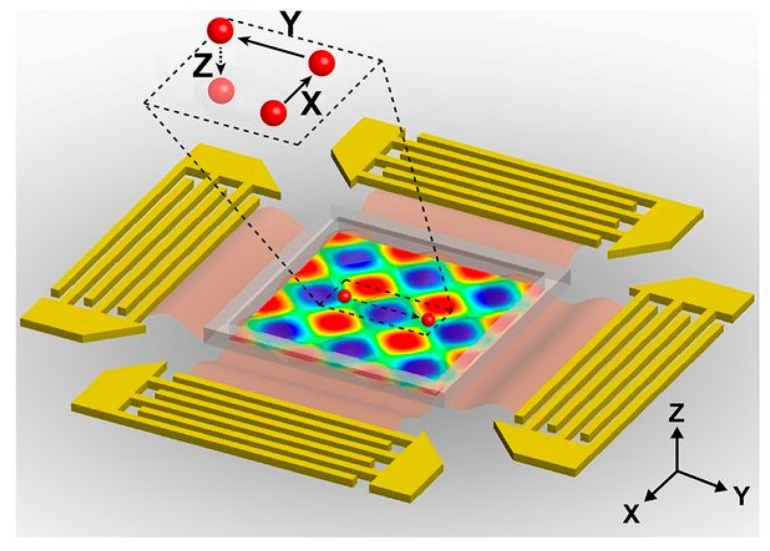
Schematic representation of 3D acoustic tweezers showing particle trapping. The solid arrows represent the movement of cell in X, Y and Z direction. The dotted arrows show an enlarged view of cell location on chip. Permission to reprint obtained from PNAS [74].

Another technique to manipulate multiple cells was demonstrated by Guo et al. They developed 3D acoustic tweezers to manipulate microparticles and cells (Figure 15). The figure shows electrodes used to create surface acoustic waves and the region of operation. The device creates standing waves by superimposing surface acoustic waves to form 3D trapping nodes. To achieve in-plane movement, they controlled the phase shift of the standing wave and the amplitude of the wave controlled the orthogonal movements [74].

## 3. Single-Cell Technologies (SCT) for Research and Diagnosis

In order to treat diseases properly, we need to understand the genetic information and metabolic pathways of abnormal cells. Efficient and sensitive detection of the chemical components within a single-cell is still challenging. In this section, we discuss some of the recently developed devices for detecting abnormal cells from a bulk of cells (Table 2).

### 3.1. Single-Cell Separation and Detection

**Figure 16:**
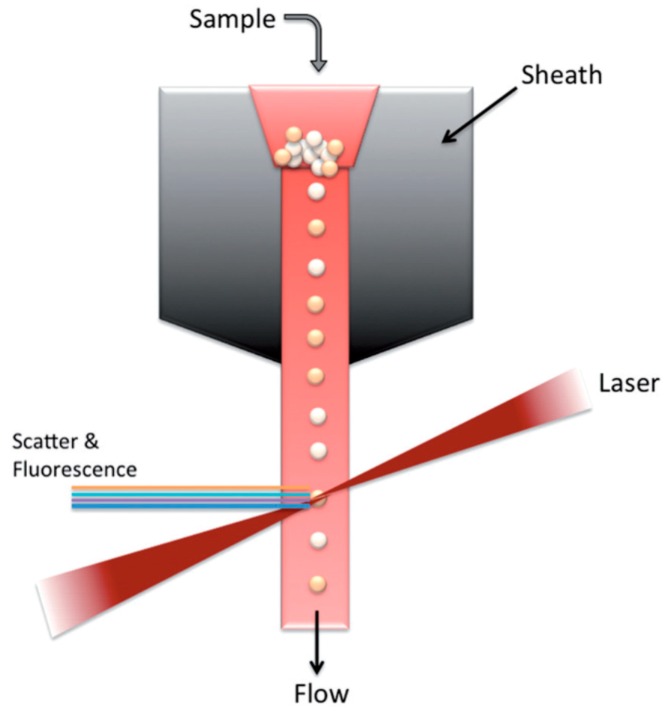
Basic Working Principle of a Flow Cytometer. “Reproduced from [99] with the permission of Taylor & Francis”.

Flow cytometry is an exceptional tool for single-cell sorting based on cell properties or fluorescence markers (1974) [96]. Fluorescence-Assisted Cell Sorting (FACS) is a type of cytometry where fluorescent-labelled cells can be separated from a heterogenous mixture of cells, on a cell-by-cell basis (Figure 16). One of the major advantages of FACS is its potential to measure multiple characteristics of cells based on the fluorescence specificities of the target constituents of the cells [97]. FACS also provides single cell data at extremely high throughput. However, DNA content analysis using FACS has shown variability in prognostic results, hence the technique is not very well accepted for DNA analysis of single cells [98].

Hence, droplet microfluidics for single-cell isolation fiber optics for target cell detection and lab-on-chip devices are combined to develop multiple cell sorting and collection through multiple channels with higher throughput [109]. Yet, for downstream single-cell analysis, the technique to separate targeted single-cells from bulk is essential. Separating cells and collecting them in 96 or 384 plate microwells is possible with FACS. However, cell sorting microfluidic devices based on antigen-antibody interactions have very high target specificity.

Fluorescent activated cell sorters and magnetically activated cell sorters to detect and separate cells are available [110,111]. However, they cannot be used directly for substrate-attached cells without disrupting their microenvironment. In addition, the lineage of cells is lost in these techniques. However, micro array-based chip technology can provide both high throughput and preservation of cell lineage. Cell detachment from a substrate in routine cell culture is generally achieved by the use of trypsin. However, extracellular surface proteins are lost in the process. A physical detachment of cells from the substrate can circumvent the loss of surface proteins. This can be useful in the downstream study of cell surface properties.

Keeping a track of cell lineage can be very useful in the study of cell differentiation and cell modification after division in diseased cells by acquiring comparative data from sister cells. Moreover, the influence of specific factors on sister cell development can be studied [112] and valuable data on the origin of genetic aberrations can be garnered [113]. However, such cell lineage tracking using conventional techniques is laborious and monotonous.

Improving on traditional dish-based approaches, the latest progress in microfluidics facilitates the monitoring and isolation of thousands of single-cells on a chip with high resolution [114,115,116,117,118]. Such approaches have allowed for the microscopical observation of single-cell self-renewal and differentiation processes [119,120]. While single-cell monitoring offers valuable information, microfluidic methods are developing numerous techniques to recover healthy individual cells for genotypic investigations, necessary for reducing complex cell rejuvenation and differentiation pathways [121]. In cell detachment procedures like poly (*N*-isopropylacrylamide) (PNIPAAm)-based detachment or trypsinization, all cells are isolated from the whole substrate without any specific cell isolation [122]. In photodegradation, photo-acid-generating cell culture substrates have lower specificity and allow improved cell release. However, this approach creates acidic by-products with potentially toxic effects and possibly disturbs cell expression and behavior [123].

Other approaches using localized trypsin or capillary vacuums have been demonstrated; though these are still restricted to open substrates [124,125]. Optical methods using infrared laser irradiation resulted in very poor cell viability owing to heat-induced cell necrosis [126]. Recently, single-cell detachment through laser-generated focused ultrasound (LGFU) was also reported [127,128]. The focused ultrasound-induced single bubble cavitational disruption (<100 μm precision) to separate target cells. These methods are also restricted to open substrates.

To overcome these drawbacks, Chen et al. developed a novel device [105,128] through which it is possible to preserve cell lineage data. Moreover, individual sister cells can be detached from the substrate without disturbing the microenvironment and can be collected for downstream analysis. Using this approach, gene expression signatures from sister cells were successfully compared and distinguishing factors were identified. The entire process was honed to consume less time and labor.

**Figure 17:**
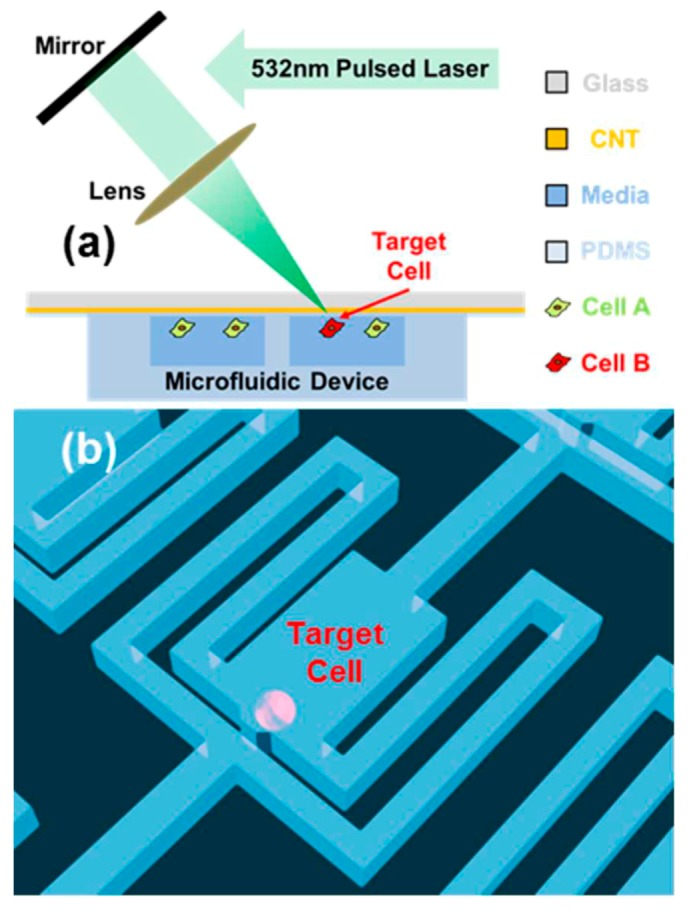
Schematic diagram of a single-cell detachment setup. (**a**) Cells were cultured in the microfluidic chamber coated with the CNT−PDMS composite. A short pulse laser is used to detach the target cell; (**b**) Microfluidic chamber and channel for cell capture and retrieval. Reprinted with permission from [105]. Copyright (2017) American Chemical Society.

Figure 17 shows the schematic view of a single-cell detachment set-up. The composite material was composed of carbon nanotubes (CNT) and PDMS. Cells were cultured on the composite film. A targeted cell was then irradiated with a single laser pulse for 7 ns and a spot diameter of 10 µm. In response to the optical energy, the CNTs act as a transducer to convert the photo irradiation into thermal energy and finally produce microbubbles. These microbubbles ruptured the PDMS film to exert a shear stress of 600 Pa on the cells seeded on top of the composite material, sufficient to induce single-cell detachment. The further expansion of the microbubble caused lateral displacement of the targeted cell. The laser beam was focused over a region of 10 µm in diameter, conducive to single-cell detachment. Furthermore, this approach can be honed to generate local detachment of a cell section.

Upon integrating the composite film with the microchannel array, the detached cells were successfully retrieved. To study the sister cells, a microchamber was fabricated with microchannels for cell retrieval.

### 3.2. Antibody-Based Single-Cell Screening

Flow cytometry is capable of detecting and sorting cells, based on their size, shape, surface proteins and fluorescence markers. Over the past three decades [129], many methods have been developed to detect circulating fetal nucleated cells (CFNC); for example, high-throughput microscopy [130,131], filtration [132], magnetic-activated cell sorting (MACS) [133,134], gradient centrifuge [135,136], fluorescence-activated cell sorting (FACS) [137,138], and microchip technologies [139,140]. These techniques can isolate individual cells for further analysis.

Liquid biopsy shows significant promise for future diagnosis. However, detection and isolation of targeted individual cells from millions of cells remains a challenge. The detection and capture of a targeted circulating cell is a step of critical significance after obtaining a liquid biopsy sample. Such an approach prevents the need for invasive biopsies that are frequently required for prognosis, therapy assessment, and proteomic and genetic studies. However, this method has drawbacks that include poor detection and separation with low cell density (one cell per billion blood cells) and heterogeneity.

#### 3.2.1. NanoVelcro Microchip

In contrast to current rare-cell sorting methodologies, “Nano Velcro” rare-cell assays may significantly improve the performance of rare-cell enrichment from blood [141,142]. By incorporating the Nano Velcro substrate with a superimposed microfluidic chaotic mixer, a Nano Velcro microchip can be produced. The microchip has been validated with high specificity and sensitivity for computing Circulating Tumor Cells (CTCs) in different solid tumors, including CTCs of lung cancer [143,144,145], kidney cancer [146], pancreatic cancer [147], and prostate cancer [148,149,150]. CTCs have been isolated by Nano Velcro microchips coated with electrospun poly (lactic-*co*-glycolic acid) (PLGA) nanofibers, coupled with a commercial laser capture microdissection (LCM) technique.

**Figure 18:**
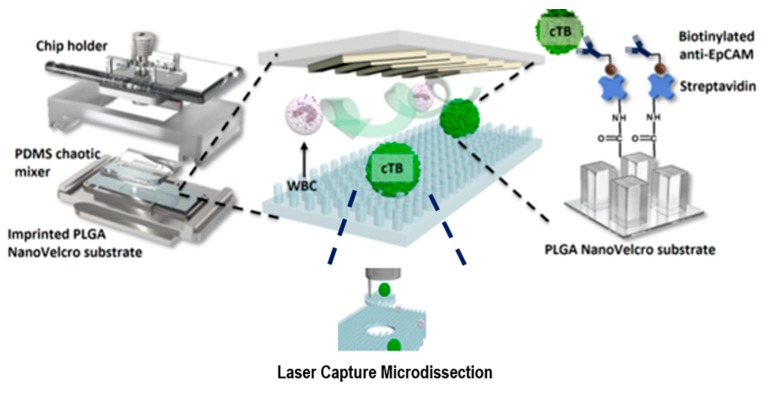
Anti-EpCAM functionalized nano Velcro microchip is assembled using a chip holder to hold together a chaotic mixer on top of an imprinted PLGA Nano Velcro substrate. “Reprinted with permission from [151]. Copyright (2017) American Chemical Society.”

The non-invasive technique for prenatal diagnosis developed by Hou et al. [151] involved the fabrication of a microchip that acted as a Nano Velcro to detect circulating fetal nucleated cells (CFNC). Figure 18 schematically illustrates the structure of the Nano Velcro microchip, which is, an array of nanopillars designed with optimum pillar dimensions. Anti–Ep Cam antibody was used to functionalize the substrate to capture CFNC in the maternal blood. After the maternal blood was gradient centrifuged for red blood cell depletion, it was passed through a chaotic mixer. The Nano Velcro and the chaotic mixer easily slide inside a metal sandwich frame, which is customized to align and hold two components together. Upon passing the sample through the chaotic mixer, within the initial three lanes itself, the CFNCs were captured in the Nano Velcro. The LCM technique was used to focus on each individual CFNC to separate each single-cell from the Nano Velcro chip for genetic testing.

#### 3.2.2. Microarray Chip Based Screening

Screening is the task of separating cells into numerous streamlines based on the distinction of their physical or chemical properties and obtain them through specific outlets. This phenomenon can be naturally observed in blood capillaries wherein red blood cells, due to their small size, flow in the central channel whereas white blood cells flow in the periphery.

**Figure 19:**
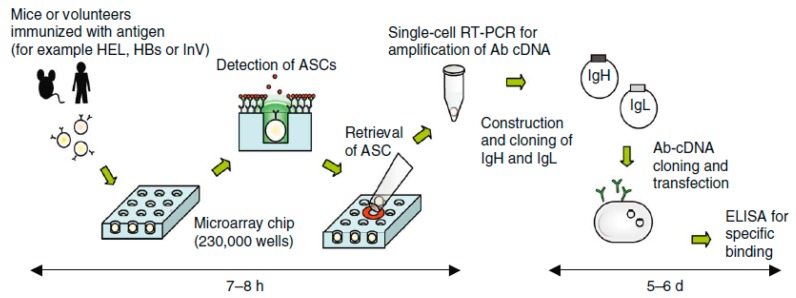
Schematic representation of the micro array-based detection and retrieval of targeted antibody secreting cells and downstream analysis. Reprinted with permission from [154]. Copyright (2009) Springer Nature.

Yamamura et al. proposed a device for efficiently screening individual active B cells from an array of lymphocytes and retrieving targeted cells (Figure 19) [152,153]. Jin et al. proposed a modified design with micro array chip for screening and separating individual cells that secrete targeted antibodies. This device uses the principle of highly sensitive and highly specific enzyme linked immunospot assay (ELISPOT) for the detection of antibody secretion. The chip has an array of hollow sites with optimized dimensions to capture individual lymphocytes. The surface of the chip is coated with immunoglobin specific antibody (IgG-Ab). These IgG-Ab trap the antibodies secreted by each individual cell around the trapping sites. On addition of targeted antibody specific fluorochrome tagged antigens, doughnut shaped fluorescent signals can be observed on the chip. Thus, targeted individual cells can be retrieved from the chip for further downstream analysis and application. This device can handle 234,000 individual cells at a time, thus proving to be a high throughput cell detection and retrieving system [154].

Development of antibodies for numerous diseases obtained from B cells is an upcoming field in therapeutics study. The antibodies for particular diseases can be obtained either by using media of cultured B cells or by gene cloning. However, this is an expensive and laborious technique. Next generation sequencing (NGS) is a useful technique wherein millions of B cells’ paired repertoires are profiled. However, it suffers from low screening throughput. In a recent advancement, Rajan et al. [102] demonstrated the use of droplet microfluidics for high throughput screening of paired repertoires. Individual B cells are captured in micro droplets along with the reagent. This allows high throughput screening, phage display and NGS.

#### 3.2.3. Micro Vortex Based Cell Isolation

**Figure 20:**
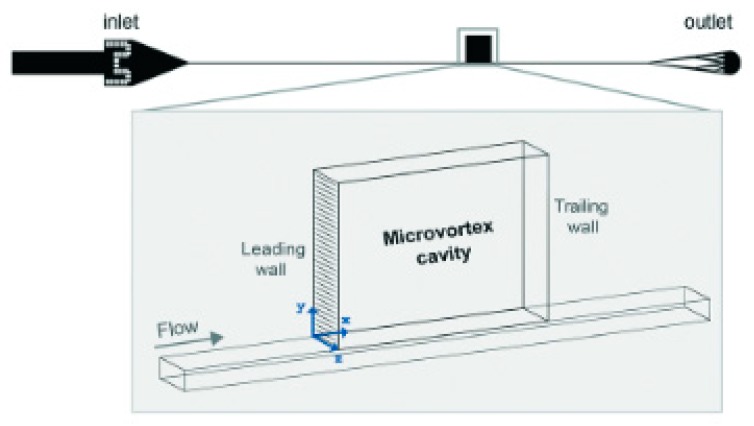
Schematic diagram of the device proposed by Khojah et al. with a micro-cavity 1 cm from the inlet. The microvortex forms in the cavity at high Reynolds number. Reproduced from [103] with permission of The Royal Society of Chemistry.

Stott et al. [155] showed the employment of microvortices to isolate rare cells from fluids. They extended the technique of mixing solutions by generating microvortices in fluidic channels via transverse flows introduced by Stroock et al. [156] to capture CTCs from whole blood. Stott et al. were able to detect rare clusters of 4–12 cells from input blood, which were not detected in the traditional CTC chip [80]. Khojah et al. [103] demonstrated the capture of different sizes of cells by tuning the fluid flow properties on chips having the same geometry under all cell inputs (Figure 20).

Trapping reservoirs of the same height but different aspect ratios were placed at a distance of 1 cm from the inlet of the rectangular flow channel. They defined three phases of flow, each varying in the range of Reynolds number of the flow in the main channel. In phase I, corresponding to 100 ≤ Re < 175, the larger particles were observed to spiral into the cavity and orbit around a vortex core which initially forms near the leading wall and moves towards the cavity’s center. Smaller particles were observed settling in large orbits, showing unstable behaviour and tendency of exiting the cavity over time. In phase II (175 ≤ Re < 225), large and small particles shared the same orbit trajectory around the vortex core. In phase III (225 ≤ Re < 300), the vortex core shifted from the center of the cavity to the trailing wall and small particles orbited closer to the vortex core, while large particles orbited away from the core and closer to the walls. Di Carlo et al. then tested the capture capacities of these three phase MCF-7 and MDA-MB-231 cells. They found phase I to be the optimal phase for MCF-7 capture and phase III to be the optimal phase for capture of MDA-MB-231 cells under different aspect ratios. Thus, the proposed system was able to capture cells of different sizes by varying the flow parameters

## 4. SCT for Therapeutics Development

Specific gene function studies within a single-cell and studies on gene response to drug molecules help scientists to understand the heterogeneity of a similar population of cells [157]. Many of these studies rely on introducing genes into cells. Several gene delivery methods have been developed, such as viral transduction, calcium phosphate precipitation, liposome, microinjection, sonoporation, electroporation, etc., [2,158,159,160,161,162,163,164]. Although none of these approaches offer all of the desired results at once, physical delivery techniques are advantageous in terms of high efficiency, high cell viability, better control, diverse cargo delivery capability, selectivity, cost-effectiveness, and reproducibility [27]. Viral transduction is a popular method used for the delivery of macromolecules. More than 90% of clinical trials being carried out are via viral transduction methods. The desired genetic material is incorporated in the genetic material of the viral vector. The viral vector is modified to suppress the disease-causing genes. This method is cell specific. The major advantage of viral transduction is that the viruses have natural mechanisms to overcome the cell membrane barrier of host cells. However, we cannot control the cargo dosage besides virus has limited genetic material carrying capability. Moreover, the viral transduction approach has immunogenic response issues and process customization is required after changing cargo and cell types [158]. In this section, we describe some of the recent techniques for therapeutics (Table 3).

### 4.1. Single-Cell Electroporation

Electroporation is a physical method used to deliver cargo into cells by creating nanopores in the cell membrane. Upon the application of an externally applied electric field, the cell membrane deforms creating membrane nanopores, when the applied voltage exceeds the cell membrane threshold voltage [172]. Single-cell electroporation was first demonstrated in 1998 when two carbon microelectrodes were placed in close proximity to a cell and an electric potential was applied between the electrodes; the cell membrane was deformed and molecules entered into the cell through diffusion [173]. Several other approaches to single-cell electroporation have been demonstrated using micropipettes [174], microfabricated devices [175] and microfluidics [176]. Longo et al. through their work created stable mammalian cell lines by transfecting them with desired cargo using electroporation [165]. Potter et al. demonstrated mammalian cell electroporation for in-vivo gene therapy in cancer treatment and DNA vaccination [177]. Even though these methods offer advantages such as high throughput and high efficiency, their application is still limited due to low cell viability [178,179].

**Figure 21:**
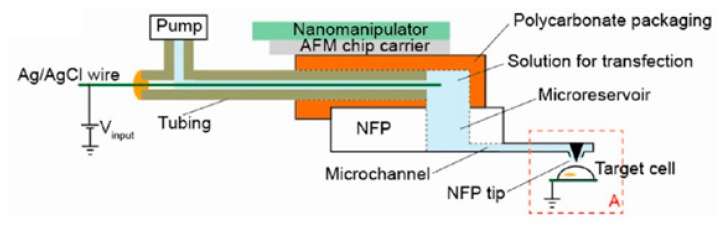
Schematic representation of the packed nano fountain probe (NFP) for single-cell electroporation. Reprinted with permission from [180]. Copyright (2013) American Chemical Society.

With a view to overcoming the low cell viability issue, Kang et al. [180] developed a technique known as nano fountain probe electroporation. Figure 21 schematically illustrates the nano fountain probe approach.

The chip is fabricated incorporating a microfluidic cargo flow channel into a cantilever with its opening at the tip. The chip has 12 microfluidic drug tube-embedded cantilevers, all connected to a common micro-reservoir. An electrical connection between the conductive wire and the conductive cell substrate generates a concentrated localized electric field at the individual cell site, creating a pore in its membrane. A drug can then enter into the cell through the capillary effect, or a micropump can be used to pump the drug into the cell. This gives better temporal resolution on the diffusive drug entry. Such a system accessorized with a micromanipulator or an AFM can deliver a drug to a precise location within the single cell and multiple single cells can be efficiently transfected as per requirement within the shorter time range. Since the inception of nano fountain probe single-cell electroporation, the voltage requirements needed to realize a successful result have been reduced from kilovolts to only a few volts. Use of a micro-reservoir prevents overconsumption of drug molecules and the system can be parallelized for high throughput applications. Drawbacks of this technique include the requirement of a conductive fluid for delivery, cell culture, and limitations in delivering large cargo into the cell.

**Figure 22:**
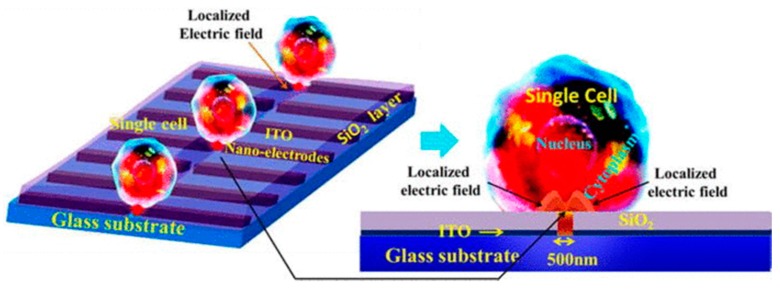
Schematic representation of localized single cell nano electroporation (LSCNEP) chip. Reproduced from [162] with the permission of AIP Publishing.

Santra et al. proposed a device for selective and localized single cell nano electroporation (LSCNEP). This device consists of electrodes having 500 nm gap, fabricated on a glass chip to reduce the affected cell membrane area and ensure single cell electroporation. An insulator acting as the passivation layer avoids direct cell contact with the electrodes. This reduces any heating effect as well as creation of any harmful ions during the process. The cell is seeded on top of the device. On application of suitable voltage, an intense electric field acts on the local region of the cell membrane as shown in Figure 22 [162]. The device is capable of achieving high throughput, precise drug delivery controllability, and high cell viability (98%) [181].

### 4.2. Optoporation

#### 4.2.1. Bulk Optoporation

One of the most promising emerging technologies for intracellular delivery is photo or optoporation, where a laser is focused onto a cell membrane in the presence or absence of metallic nanoparticles to produce micro or nanobubbles, which disrupt the cell membrane and produce transient membrane pores to deliver molecules into cells [182]. Membrane permeability can be increased either by using a tightly focused laser beam (pulsed or continuous wave laser) or by a broad laser beam in combination with sensitizing nanoparticles [183]. In 1984, Tsukakoshi et al. reported an optoporation technique to deliver membrane impermeable molecules into a cell by creating pores using a pulsed laser [184]. Nikolskaya et al. used a continuous laser to deliver fluorescent molecules in neonatal rat cardiac cells. This study demonstrates high target specific delivery and high spatial resolution enabled by optoporation [185]. Andrew et al. used femtosecond pulsed laser for optoporating Chinese hamster ovarian cells [186]. Use of pulsed lasers over continuous lasers for optoporation gives better energy efficiency and reduces cell death.

In photothermal delivery, metal nanoparticles are used to create Localized Surface Plasmon Resonance (LSPR). The laser electric field generated is enhanced due to the lightening-rod effect. As a result, plasmonic bubbles are created in the surrounding medium, which generates shock waves. These shock waves disrupt the cell membrane to create transient pores [31]. Bastein et al. studied the visible and near-infrared laser wavelengths and their effects on intracellular delivery and cell viability after exposing mammalian cells. The study proved that the use of near-infrared wavelength can achieve better cell viability [187]. Ranhua Xiong et al. showed that the optoporation efficiency in presence of metal particles is higher as compared to direct laser exposure for live Hela cells [188]. However, nanoparticle-mediated intracellular delivery is limited to certain sizes and types of molecules, and is only suitable for specific cell-types [189]. All these approaches come under bulk level optical transfection.

#### 4.2.2. Focused Laser Beam Based Single-Cell Optoporation

**Figure 23:**
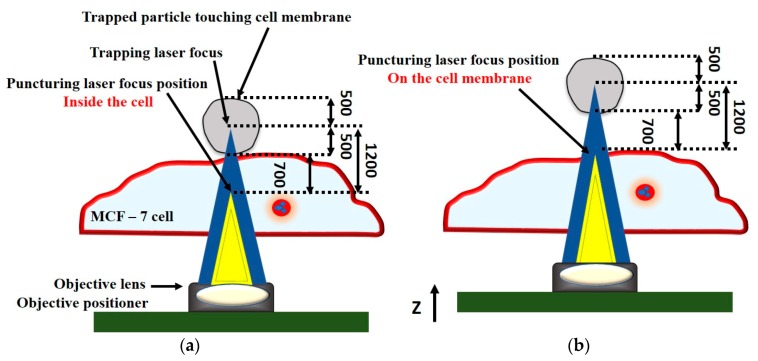
Schematic representation of particle intracellular delivery. (**a**) 1064 nm laser (blue) is used as an optical tweezer to trap particle. (**b**) An 800-nm femtosecond laser (yellow) is used as a puncturing laser to create pore on cell membrane. Redrawn from [166].

Waleed et al. reported single cell transfection of MCF-7 cells with pAcGFP1-C1 plasmid coated 1 µm amino-based polystyrene microparticles using a femtosecond laser (800 nm) in combination with optical tweezers (1064 nm) (Figure 23). This technique offered control over the amount of plasmid that is inserted into a cell, high selectivity, and aseptic conditions due to the non-contact nature of the technique. The main limitations of this technique are the low transfection efficiency of 12.7% and the low transfection frequency of 20 cells per hour [166], which makes it not feasible for practical applications.

#### 4.2.3. Metal Nanostructure Assisted Single-Cell Optoporation

**Figure 24:**
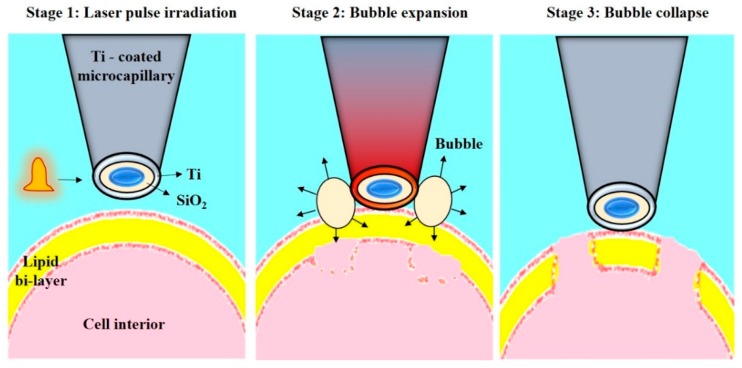
Schematic representation of the Nano blade. The figure depicts the process of cell membrane pore formation by creating micro bubbles. Redrawn from [190].

Wu et al. reported another single cell transfection technique based on metal light interaction based nano bubble, which created shear stress on cell membrane (Figure 24). They fabricated a nano blade by depositing 100-nm titanium on glass micropipette. This nano blade is held near the cell surface. Pulsed laser is irradiated on the titanium nano structure. As a result of LSPR, micro bubbles are created, which disrupt the cell membrane to create transient pores [190]. Using this technique, they pumped the cargo into the cell through a micropipette with 46% transfection efficiency and 90% cell viability [167].

### 4.3. Single-Cell Mechanoporation

**Figure 25:**
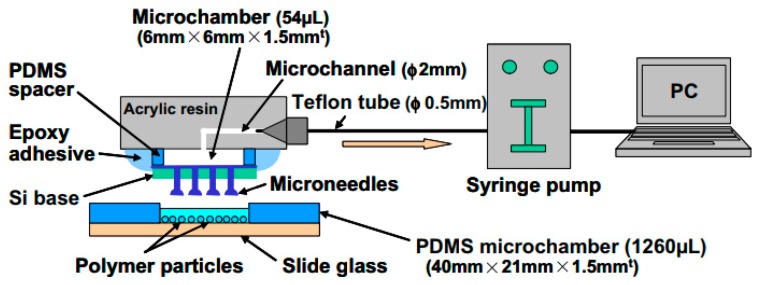
Overview of the system designed by Shibata et al. “Reprinted with permission from [193]”.

This technique uses physical force to penetrate the cell membrane and deliver the drug inside the cells. Microinjection is an example of mechanoporation. A microneedle is used to inject the drug into the cytosol. A micromanipulator is used to hold the microneedle and control the pressure for cargo delivery. This technique allows high delivery efficiency and a throughput of about 1000 cells per hour. This system is further upgraded in the development of microfluidic devices with stationary needle probes and flowing cells to make the system more robust and increase throughput [27]. Thus, hollow engineered probes such as hollow micro-capillary arrays [168,169], hollow nanoneedle arrays [191] and carbon nanosyringe arrays [192] have been fabricated on the cellular scale. To achieve better penetration of the cell membrane and successive cellular injection, the wall thickness of the hollow probes is reduced. Limited efforts have been made to develop a microprobe array for single-cell manipulation. In particular, to reduce the stress concentration at the cell, Shibata et al. [193] fabricated a hollow microprobe array with hemispherical tips (Figure 25). This device is a microarray chip, designed for high-throughput intracellular delivery of single-cells that even allows for the manipulation of different types of single-cells in 3-D and parallel formats. For fabrication simplicity, only a single photomask was used to design the probes in previous studies [194]. The fabrication process established hitherto lacks approaches for monitoring the inner diameter, outer diameter and a wall thickness of the probes. It is essential to improve the fabrication process to control the probe dimensions and to optimize the structures used for single-cell manipulation.

**Figure 26:**
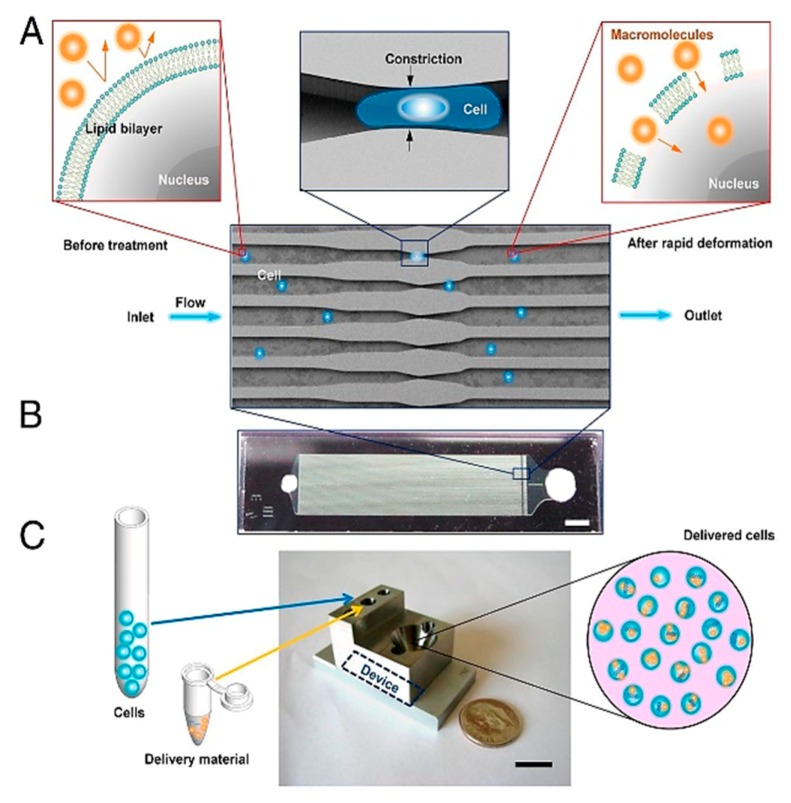
(**A**) Mechanism of formation of transient pores in cells on passing through the microconstrictions in the device designed by Sharei et al.; (**B**) Image of the finished device with a zoomed-in view showing the cell flow channels with the microconstrictions. (**C**) Image of the device packaging. “Permission to reprint obtained from PNAS [170]”.

Recently, devices that used constrictions to apply physical stresses to cells for enabling drug delivery into cells have gained prominence in the field of mechanoporation. Sharei et al. [170] proposed the first constrictions-based device for drug delivery, which forces cells to pass through constrictions in series or parallel, causing transient pores to develop in the cell membranes during this process (Figure 26). The fluid surrounding the cells are loaded with drugs, which diffuse into the cells through these transient pores. This device showed a very high throughput rate of 20,000 cells/s and delivery efficiency between 70–90%. Szeto et al. [171] used the commercial Cell Squeeze platform, based on the device described by Sharei et al., to verify the delivery of drugs directly into the cytosol of cells and not endosomal compartments through various experiments. Szeto et al. also conducted experiments to optimize the device parameters for maximizing cell viability and delivery efficiency. They used B-cells for their experiments and reported cell viability above 95% after treatment with the platform along with more than 25-fold increase in internalization of desired particles into the B-cells compared to control conditions.

## 5. SCT Analytical Strategies

Substantial progress in nanotechnology over the last two decades has greatly improved sensitivity of analysis at an individual cell level. Here, we present some of the recent methods reported for analyzing a single-cell, including cell tracking software, along with their working principles. The design and application of several devices that are highly effective in analyzing intracellular molecules and cell growth rates will also be discussed (Table 4).

### 5.1. Better Visualization of Single-Cell Differentiation

Microscopic monitoring of a single-cell from a mass of cells for the entire duration of an experiment is challenging, partly because cells can divide and migrate rapidly. To recognize and track targeted cells, only a few microscope setups, established under vibration-free conditions, are available, which offer a combination of high-resolution micro-displacement stages with supporting software.

**Figure 27:**
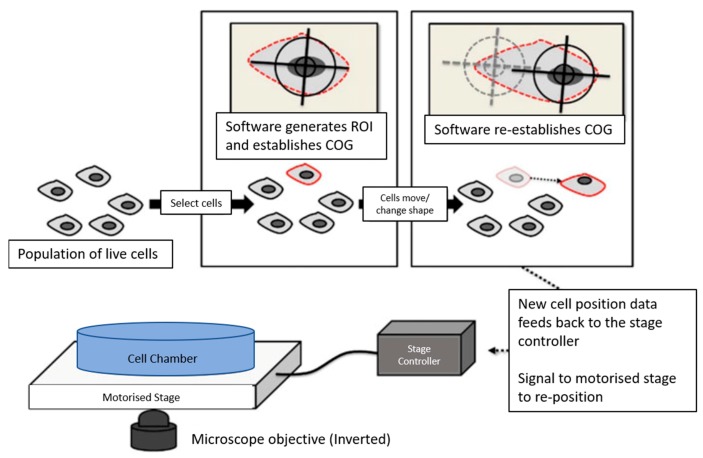
Tracking of individual cells from a population of live culturing cells using a live-cell imaging microscopic platform with a high-resolution micro-displacement motorized stage. “Reprinted with permission from [202]. Copyright (2016) Springer Nature”.

A number of proprietary software packages are available that share a similar strategic approach to cell tracking. In this approach, the region of interest is first selected by the operator using the software. The algorithm identifies the center of the cell and thus establishes a set of features for tracking the translocation of the center of a cell and its morphological change along subsequent frames. The micrometer-scale resolution stage is repositioned to the new location of the cell as soon as a large movement takes place beyond the starting position. As long as the *X*–*Y* stage facilitates micrometer level adjustments, a cell can be reliably tracked. In addition to such *X*–*Y* stage displacement, most modern systems allow for fine-tuning of the *Z*-axis to investigate cells suspended above the focal plane. Sub-micron adjustment of the *Z*-axis allows for the selection of vertical optical slices, thus enabling spatiotemporal 4D recording of cells for rendering and modeling [27]. Figure 27 schematically illustrates the tracking of individual cells from a population of live cells.

A software named BaSiC has been used to observe obscure changes involved in cell development. It can successfully detect transcription factors used by cells during decisive developmental steps. Such detection requires prolonged tracking of cells with background alterations and shading, which is tedious using traditional tools. However, BaSiC has an inbuilt background alteration correction algorithm, which is an exceptional tool for the study of early-onset transcriptional factors and is especially useful in stem cell studies. One of the key features of BaSiC is the use of the L1 norm for error measurement, which leads to faster convergence, thus requiring fewer images for processing compared to other techniques. This is especially appreciable in situations where only a limited number of images are available. Using the L1 norm also allows for sparser solutions, thus accommodating outliers such as noise better than other techniques. BaSiC also uses a spatially constant baseline signal B_i_ to account for varying background in subsequent images. Using this tool, developers observed hematopoietic stem cell differentiation. The results could visibly differentiate between the up-regulation of a certain transcription factor in one of its mature cell lineages which remained dormant in its other type of mature cell lineage, providing information on decisive factors in cell development [195].

Another software that deals with the issue of uneven illumination without requiring any reference images is CIDRE (correction intensity distributions using regularized energy minimization), introduced by Kevin Smith et al. [203] A predecessor to BaSiC, CIDRE too uses both the flat-field and dark-field to stitch images. One of the major assumptions of CIDRE is that the input images are uncorrelated. CIDRE matches the performance of BaSiC for larger collection of images. CIDRE estimates the zero light or dark light term *z* and the illumination gain *v* at all points simultaneously using an energy minimization technique [204]. The method models distortions to images by the following equation:

$$I\left(x\right)=\frac{I_{0}\left(x\right)-z\left(x\right)}{v\left(x\right)}$$

where *I*(*x*) refers to the true image and *I*_0_(*x*) refers to the image captured by the sensor. Since *v* and *z* are already determined by the method as described above, the true image is extracted using this equation.

### 5.2. Quantifying Single-Cell Growth

**Figure 28:**
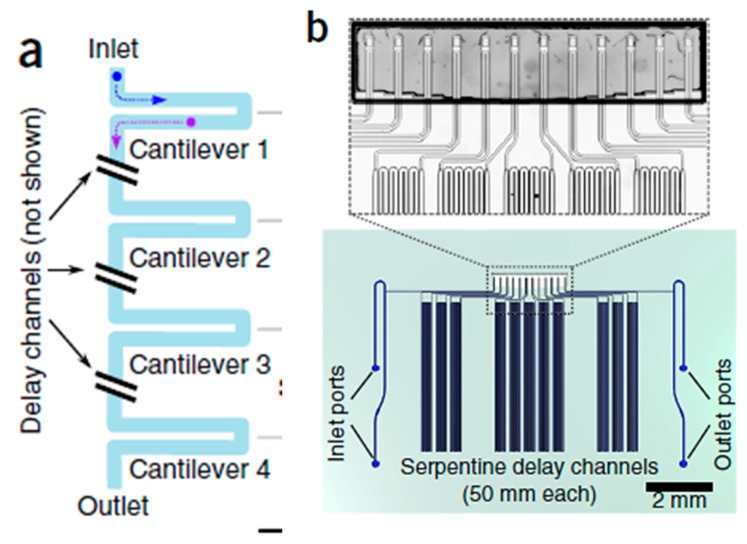
(**a**) Series of SMRs (cantilever mass sensors) separated by delay channels; (**b**) Rendering of a large channel serial SMR array device showing delay channels and the cantilevers (magnified in inset micrograph). Reprinted with permission from [196]. Copyright (2016) Springer Nature.

The growth rate of a cell is governed by a combination of several factors. Even genetically identical cells can have different growth rates due to different combinations of intrinsic molecular noise and various deterministic behavioral programs [205,206,207,208,209]. Despite having important consequences for human health, this variation is less observable via population-based growth assays. Recently, a number of methods have been proposed for measuring single-cell growth. One of these techniques involves resonating MEMS structures where a low concentration cell suspension is allowed to flow through sealed microchannels. These microchannels are etched along the cantilever length. The cantilever itself is suspended in a vacuum cavity. The principle of the operation is a change in the natural frequency of the cantilever resonator on pumping the cell suspension through the microchannel (with respect to pumping suspension without cells). Gobin et al. used a suspended microchannel resonator (SMR) along with a picoliter scale microfluidic control to measure mass and determine the instantaneous growth rates of an individual cell [210]. Son et al. developed a microfluidic system, which can measure single-cell mass and cell-cycle progression simultaneously over multiple generations [211]. Another SMR chip designed by Burg et al. consists of a sealed microfluidic channel, which is placed inside a cantilever resonator. This cantilever is housed in an on-chip vacuum cavity. When a cell suspension flows through the interior of the cantilever, it transiently changes the cantilever’s resonant frequency in proportion to the mass of the cell. These methods measure the growth of a single-cell with high precision at the cost of throughput. To overcome this limitation, a serial array of such micro cantilevers containing intermediate delay channels was presented by Cermak et al. [196]. Figure 28 shows the SMR and its working principle.

The intermediate delay allows for the essential time-span required for cell division, which can be quantified in the microchannel embedded cantilever region. Thus, high precision using the single suspended microchannel resonator (SMR) can be achieved. Growth rates of over 1 cells/min can be obtained using this technique. Observation of variations in individual cell growth is a very important aspect of cancer studies as well as degenerative disease research. This approach can give important information on drug efficacy in terms of cell growth [196].

An important factor along with growth rate quantification is cell cycle inheritance. Sandler et al. [209] developed a model for cell cycle inheritance using microfluidic flow to deposit them into microwells, observing them using time-lapse microscopy. They developed a ‘kicked cell cycle’ model to explain the correlation observed between cousin cells; there seemed to be close to zero correlation between mother-daughter cells. The main feature in this model was the deterministic influence of the circadian phase at birth on the cell cycle duration [212]. They were successfully able to validate the observed correlations with their model, thus demonstrating the importance of a non-linear process, such as the circadian clock [213], on the determination of cell cycle variability.

### 5.3. Single-Cells in 3D View

Vast technical improvements in microscopical techniques have been made over recent decades to increase the contrast between signal and background. High-resolution fluorescence microscopic technologies are highly effective in understanding the molecular-level details that underpin living organism processes. The large spectral range of available fluorophores allows for simultaneous imaging of different cellular, subcellular, or molecular components. One important contribution to the development of SCT is whole cell 4Pi single-molecule switching nanoscopy (W-4PiSMSN), with three-dimensional individual cell imaging capability [214]. W-4PiSMSN can provide 10–20 nm axial resolution in the volumetric reconstruction of 10 µm thick samples. Cell sections which previously could only be visualized by electron microscopy could now be studied by W-4PiSMSN. This overcomes the electron microscopy associated drawback in terms of the ability to study the localization of proteins within a cell. The group successfully reconstructed Golgi complex-associated COPI vesicles, endoplasmic reticulum, nuclear pore complexes and cell cilia of COS-7 cells and icosahedra shaped capsids in bacteriophages.

### 5.4. Cell Membrane Potential Measurement

Scientists often strive to study the membrane potential change occurring within individual cells due to ion exchange performed during cell signaling. Techniques such as patch clamp, planar patch clamp, and their subtypes have been used to study ion exchange during intercellular signaling. However, such techniques are unable to preserve the cellular microenvironment during measurements. Furthermore, artificial stimulations need to be applied to the cell, typically using a micropipette, prior to measurement of its response. The drawback lies in the disturbance produced in the cytosol due to dilution with micropipette contents. In order to overcome these drawbacks, nanowire devices have been developed to measure intracellular potential with nominal invasiveness. These devices can measure the potential of one individual cell at a time, as well as a network of cells simultaneously.

**Figure 29:**
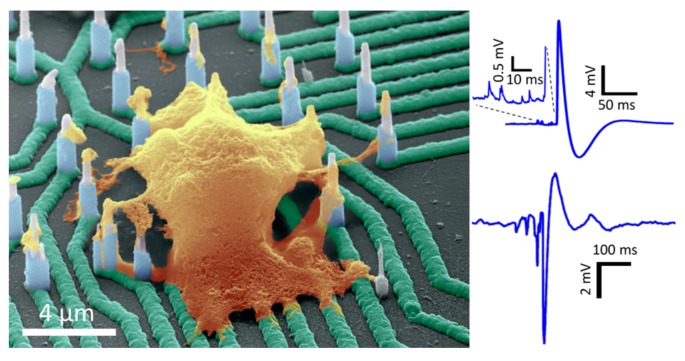
The electrical circuit model of the electro-neural system starts with the cell culture (yellow) interfacing with the electrode, all the way to the read-out electronics represented by the amplifier block (green). Reprinted with permission from [197]. Copyright (2017) American Chemical Society.

One such device was developed by Liu et al. [197] (Figure 29); it employs an electric circuit model of the electro neuronal system, which has multiple electrodes connected to a single cell. These electrodes can measure the intracellular potential at multiple locations within a single cell. Moreover, it has the exceptional capability of uniquely controlling each individual electrode.

Each electrode is 10 µm in length and 200 nm in diameter. The electrodes are spaced at a distance of 4 µm from each other. The fabrication process is uniquely adapted to avoid any electrochemical interactions between the cell and the chip at any undesirable points except for the electrode tip required for cell contact. This allows precise control when obtaining a localized signal from intracellular locations. Low contact resistance and high input impedance are achieved to avoid any errors due to noise interference. This nanowire chip has been successfully used to measure sub-threshold postsynaptic potential in human-induced pluripotent stem cell-derived neurons in mouse and rat primary neurons with high signal to noise ratio [197]. The study was performed on the same cells in vitro over a period of six weeks with excellent neuronal activity preserved in human-induced pluripotent stem cell-derived neurons. The chip was proven capable of recording small quantity neurotransmitter variations with signal precision from an individual cell in a network of neuronal cells. Obtaining such data is vital to the study of pre-synaptic and post-synaptic disorders. It can also be useful in modeling any changes in potential frequency or magnitude of diseased conditions over normal conditions [197].

### 5.5. Cytometry

Several methods are available for single-cell analysis, including laser induced fluorescence, electrochemical detection (ECD) or mass spectrometry. These methods, accompanied by capillary electrophoresis, have attracted great attention for their ability to analyze low concentrations of biomolecules [215,216,217]. Zheng et al. developed a lactate sensor with a nanoscale optical fiber to detect extracellular lactate in cancer cells [218]. However, these methods are laborious in estimating the fluorescence intensity outside and inside living cells. Matrix Assisted Laser Desorption/Ionization-Mass Spectrometry (MALDI-MS) analysis of single peptide-containing vesicles was established by Li et al. [200], but the molecular weight of the detected biomolecules was restricted to 500 to 8000 Da. Electrochemical electrodes coupled with sampling capillaries for sampling very low concentrations of mammalian cells was developed by Woods et al. [219]. By means of a capillary-microfluidic device, Omiatek et al. [220] describe cell separation, lysis, and electrochemically measured the contents of single vesicles.

**Figure 30:**
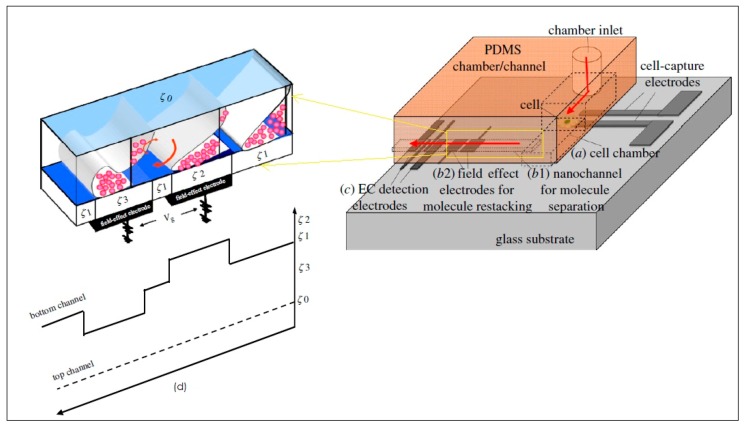
Schematic representation of a Nano-CEEC chip designed for living single-cell analysis. Step 1: (**a**) cell loading, capturing and culturing in the cell chamber; Step 2: (**b1**,**b2**) DAEKF (dual-asymmetry electrokinetic flow) separation, including sample collection, nanoseparation and restacking in the nanochannel; Step 3: (**c**) amperometric detection by the EC detection electrodes; (**d**) Schematics showing the flow-field evolution for DAEKF electrophoresis manipulated by surface zeta potentials in the nanochannel (solid arrows represent the analyte flow direction) Reproduced from [221] with permission of The Royal Society.

Another technique developed by Wu et al. [221] is capable of zeptomole-level detection of neurotransmitters released from single living cells using nanocapillary electrophoretic electrochemical (Nano-CEEC) chips. The chip is comprised of three units, a pair of targeting electrodes for capturing single-cells, a dual asymmetry electrokinetic flow device for sample collection, pre-concentration and separation, and an amperometric sensor for the detection and analysis of neurotransmitters. Figure 30 schematically illustrates the nano-CEEC chip for living single-cell analysis.

The entire process can be completed in 15 min, right from cell sampling to neurotransmitter detection. These devices might be potentially applicable for disease diagnosis.

**Figure 31:**
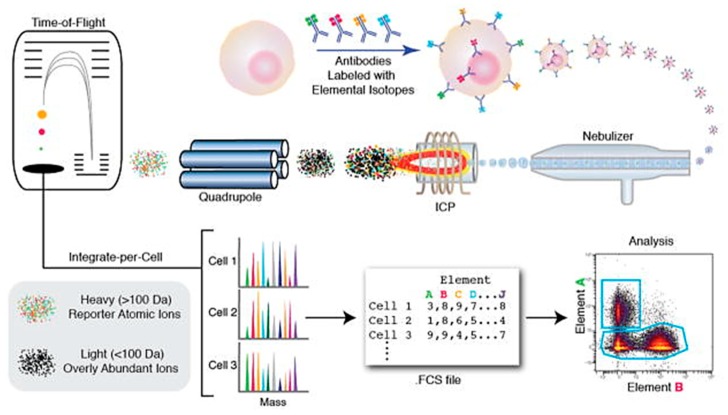
Schematic representation of the working principle of CyTOF. Reprinted with permission from [224].

Cytometry Time of Flight (CyTOF) is an advanced flow cytometry technique that overcomes the spectral overlap issue involved in studying multiple parameters with FACS. This technique allows the study of around 42 parameters simultaneously. Heavy metal isotopes are first attached to the antibodies for trapping targeted cells [222]. Once the targeted cell antigens are detected by antibodies, single cells are encapsulated in droplets by a nebulizer (Figure 31). These encapsulated cells are then passed through an inductively coupled plasma (ICP), which vaporizes them into an ion cloud. The Quadrupole Time of Flight (qTOF) mass spectrometry technique is used to measure mass to charge ratio, which can provide the spectra for tagged metal atoms [223]. Thus, the antibody-metal conjugation data along with the spectrum obtained by qTOF can provide cell marker analysis. Kay et al. reported analysis of natural killer cells by using the CyTOF technique. Huge diversity is observed in the expression of surface and intracellular markers expressed by natural killer cells based on the targeted disease. Such variability requires a large number of probes for analysis, which is made possible by the CyTOF technique [201]. However, CyTOF has lower throughput (1000 cells/s) as compared to FACS.

Erik Gerdtsson et al. [225] showed that the integration of Imaging Mass Cytometry (IMC) using metal-labeled antibodies into the existing high definition single-cell analysis [226] allowed the CTC profiling to be extended to include lot more protein biomarkers. The IMC process involves the coupling of laser ablation with single-cell mass cytometry performed using the CyTOF technology [227]. They reported a multiplexing level of 40 proteins, which can be used for the generation of informative biomarker panels. Thus, it shows high potential for complete characterization of blood samples and extraction of crucial biomarkers for assessing the liquid phase of cancer.

### 5.6. Single Cell Sequencing

Next Generation sequencing (NGS) is a sequencing technology that enables the sequencing of entire human genome within a day. In this technique, the entire genome is first divided into millions of fragments and sequenced in parallel. The data obtained from individual fragments are then mapped to a reference human genome via bioinformatics analysis. This process is repeated multiple times to get accurate data on any DNA mutation. NGS can also be used for extracting data from targeted genetic locations. It can be used for detecting novel diseases, as it interrogates each individual fragment without any prior knowledge of the gene loci. NGS has better sensitivity towards low-frequency variants due to its high sequencing depth [228]. NGS along with drop-seq technique has many applications in DNAseq, RNAseq and ATACseq (chromatin) of multiple individual cells. In RNAseq, mRNA is reverse transcribed to DNA, which is further used as the substrate for analysing the cells’ state based on RNA molecule expression profile. This can also be used for proteomic studies. Gawad et al. explained the significance of single-cell genomic analysis in understanding intratumor cell heterogeneity. Such analysis can provide dynamic data on the process of cancer development [229]. Buenrostro et al. showed that analysis of the single cell epigenome could be done quick enough to enable users to make better clinical decisions [230]. Some of the major technologies developed recently also include RNA-Seq to study gene expression in bulk tissues, specifically single-cell RNA-Seq done by Wu et al. [231]. This single-cell technique was shown to be quantitatively comparable to multiplexed quantitative PCR (qPCR) [232]. Another advancement in this technology is the development of highly multiplexed single-cell qPCR introduced by Vanlnsberghe et al. This device integrates all the steps for qPCR, including cell detection, isolation, and lysis for the measurement of qPCR reactions of 200 single cells in parallel. The data obtained revealed cell-to-cell heterogeneity for two cell types following cell differentiation [233]. The major difference between RNA-seq and qPCR is that the former allows a broader scope of gene analysis at lower sensitivity, which is the opposite for the qPCR technology. 

### 5.7. Intracellular Access

The study of intracellular proteins and mRNA is a pivotal step in the study of pathogenesis. Many highly sensitive single-cell mRNA and protein detection methods, such as those based on phenotype heterogeneity and noise in cellular systems, have been developed [229,231]. These methods rely on lysing the cells to extract the intracellular contents for analysis. This limitation is particularly problematic when studying dynamic transformations. To overcome the limitation, many minimally invasive sampling devices have been designed to access the interior of cell matrices. One approach inserted atomic force microscope tips through a cell surface to make direct contact with the cytoplasm of living cells with high positional accuracy. This method was used to quantify mRNA expression in a single living cell without causing severe damage to the cell [232]. Saha-Shah et al. used nano-pipettes (diameters < 1 μm) to collect nano-liter volumes (<10 nL) of cell-matrix samples from single *Allium Cepa* cells [233]. However, this technique may impact cell survival and limit cell studies to cells of relatively large sizes. A recent development by Cao et al. [198] has studied the limitations involved with lysing cells in order to extract its contents.

**Figure 32:**
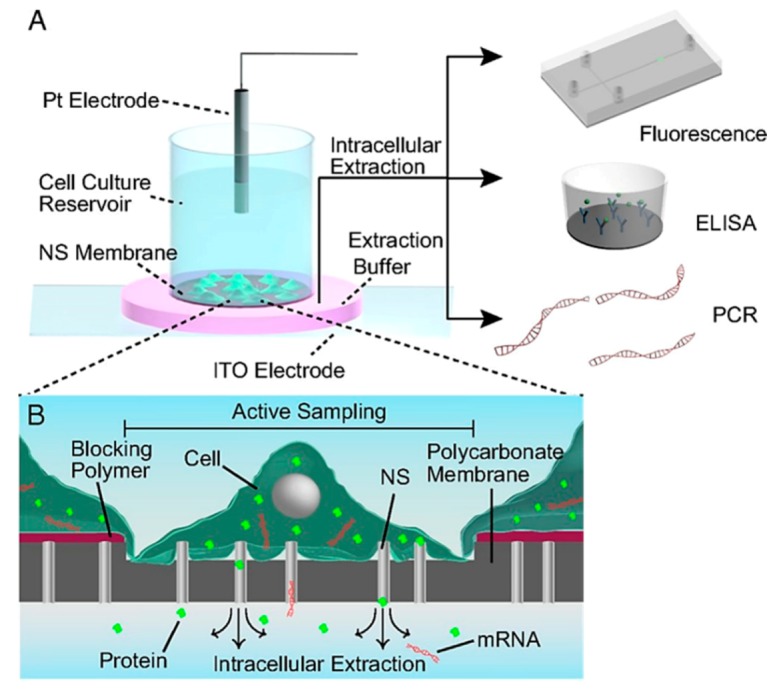
Design and operation of the NEX (Nanostraw Extraction) sampling system. (**A**) The system consists of a polymer membrane with protruding 150-nm diameter nanostraw (NS) attached to the bottom of a cell-culture dish. Sampling is performed by temporarily electroporating the cells cultured on the NS, allowing cellular content to diffuse through the NS and into the underlying extraction buffer (pink). An aliquot of the buffer is then aspirated with a standard pipette and analyzed conventionally, using fluorescence imaging, ELISA, or qPCR; (**B**) Schematic representation of the extraction process. Permission to reprint obtained from PNAS [198].

Figure 32 shows the schematic representation of nanostraws and their operations. The chip has nanostraws embedded vertically in the cell culture substrate. On applying a bulk electric field, nanopores are created on cells. The intracellular contents drain out through the nanostraws into a buffer medium by diffusion. The nanostraws being 150 nm in diameter can be used for small molecules, proteins and mRNA extraction through nanopores existing for longer durations without cytosolic leakage. It provides a dynamic non-destructive intracellular sampling environment to study the same cell or set of cells throughout its cell cycle [198].

## 6. Development, Challenges, Applications and Future Prospects of Single-cell Technologies

The entire article provides a compact outlook to the reader about the stages involved in SCT development for different cellular characterization, disease analysis, etc. Manipulation of multiple cells and their behavior are essential to understand the in-vivo environment to reduce possible errors. The corresponding tools are continuously being developed; such as optical tweezer (1988) [234], manual micropipette (2001) [235], automated micropipette (2016) [236,237,238], manual pump based parallel micropipette array (2015) [70], etc. The optical tweezer was first introduced to trap bacterial cells in 1988 using an infrared laser source [236]. A decade later, dielectrophoretic tweezers were used for cell handling within microfluidic channels [239,240,241]. In 2000 [242,243,244,245], optical tweezers showed wide applications in the study of physical properties of cell, protein and DNA interactions. However, dielectrophoretic tweezers did not show such rapid development. Techniques such as rapid electrokinetic patterning (REP, 2008) and optoelectrokinetic tweezers (OET,) emerged in 2008 and 2013 [246,247,248]. As compared to single optical trap, multiple optical traps from single laser source were demonstrated recently in 2017. Compared to the recent developments in these techniques, microfluidic-based microarray probe shows a lack of cell selection flexibility, although it has the capability to increase throughput. However, the technique of 3D optical traps has lower throughput and is more expensive than microfluidic technique. Nevertheless, both dielectrophoresis and acoustic 3D trap allow in-situ single-cell handling. However, single-cell manipulation is a complex task and needs to overcome various challenges (such as increasing throughput and experimental repeatability for robust design flexibility) in control over cells and tools that can allow handling of many isolated cells simultaneously with high specificity.

Throughout this article, we have discussed lab-on-chip, microfluidic Bio-MEMS, and µTAS platform for cellular characterization and analysis. We interpret that the scope lies in the development of different tools and their applications. There is a wide gap in the availability of the current technology and requirement of actual tools. For example, antibody-antigen reactions are currently operated at a bulk level, which may conceal the low-frequency information of migrating cancer cells. Another problem is the incubation time required for these tests. Diagnostic tests have other limitations as well, which include, among others, the availability of appropriate reagents at all times. Hence, the applications of lab-on-a-chip technologies have garnered interest in overcoming these problems for precise diagnosis, and have been found to be the origin of most applications for single-cell technology development. This area of research needs more attention in the future.

Cell isolation, sorting, detection and analysis hold great promise for disease diagnosis. Blood is a medium of transport for various cells throughout the body. Thus, liquid biopsy can potentially provide relevant information for any disease. A few of the major challenges faced in this field are low targeted cell concentration, specific antibodies for capturing targeted cells, and unwanted cellular interference. Another important obstruction is lack of spatio-temporal context due to which downstream analysis becomes impossible [100]. There is huge diversity in omics analysis, making it more challenging for direct detection or expression of targeted genetic materials. Further, omics analysis requires high sample purity in adequate quantities. Among omics technologies, proteomics proves to be much more complex than genetic material analysis, mainly due to the lack of protein amplification techniques and lack of highly specific affinity probes [249]. The entire process is a combination of various steps, making it an interdisciplinary task. The techniques that we discussed in the article pave the way for a solution to some of the above challenges.

Cells manipulation and drug delivery through transient hydrophilic membrane pores involve sophisticated methods. In the future, modification and control of cell environment should be enabled such that cells can behave normally. Thus, regulating the cell microenvironment along with cell treatment can be the appropriate perspective towards treatment. It is necessary to understand the cause and changes in cell microenvironments, which is different for different cell individuals. Hence, the same medications do not have the same effect on different individuals. Thus, the requirement of individual medication arises. Endoscopy tools can be modified and incorporated with the lab-on-chip device, to analyze the cancer microenvironment and compare with the non-cancer environment of the same individual to understand the imbalance. Since a chemical imbalance is only a potent form of energy imbalance within the body, manipulation tools are necessary at every stage of this process. The packaging of required setup into a compact form is essential for an effective and efficient manipulation.

**Figure 33:**
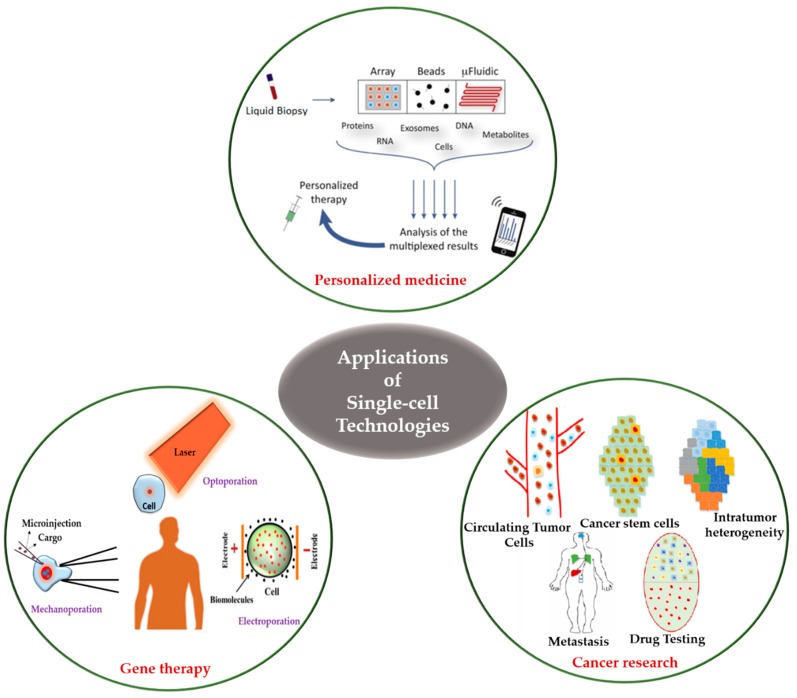
Applications of single-cell technologies. Reprinted with permission from [29,250].

Figure 33 shows the schematic representation of applications of single-cell technologies. Multiplexed point of care (xPOCT) is an emerging field for personalized medicine. Precise and early diagnosis is the critical step towards treatment of any disease. Prescription of treatment for diseases based on single biomarkers obtained through screening can be ambiguous. Hence, we need techniques that are highly sensitive in detecting multiple biomarkers for a diseased condition. Towards this purpose, Bio-MEMS devices are already available and show a lot of potential for advancement. The advantages of Bio-MEMS devices include easy-to-use non-intervention-based systems, low sample requirement, and highly accurate results, compared to bulk pathological techniques. Thus, incorporating single cell technology chips is a prospective advancement in xPOCT [250].

SCT also has wide applications for cellular therapeutic development. We introduced the most popular and upcoming physical methods, such as electroporation, optoporation, and mechanoporation, for cellular therapy and analysis. While other techniques (such as sonoporation [251], gene gun [252] and magnetoporation are also available [253]), they suffer from a lack of control for single-cell transfection (with the exception of electroporation and photoporation). Another important tool for delivery is microinjection, which offers [254] low throughput, low repeatability, and lack of robustness. Electroporation has been used for creating transient cell membrane pores while electrophoresis enhances movement of nanoscale-sized charged particles into cells [255]. While constrictions-based devices are still novel in the field of mechanoporation, they show a lot of promise, owing to their high transfection rates and ability to successfully deliver tough cargo such as CRISPR-Cas9 complexes into cells [256]. These devices report a higher cell viability rate after treatment in comparison to other physical therapeutic methods such as electroporation. Furthermore, Ding et al. designed a device incorporating both electroporation and mechanoporation for nuclear transfection of DNA into mammalian cells, thus introducing a strong precedent for hybrid devices in the future [257]. We can expect to see more devices combining different transfection techniques in advantageous ways to achieve transfection of tough cargo like never before.

Over the last two decades, techniques for single-cell imaging have shown tremendous development. Integrating it with various manipulation technologies can allow improvement of cellular diagnostic and therapeutic applications. In addition, real-time monitoring techniques have made imaging-based analysis convenient.

SCT has contributed to the development of many potent tools to detect cancer cells, illuminate epigenetic variations, analyze, and personalize treatment, among others. Such information on epigenetic variations can provide important information on neuronal degenerative diseases, such as Alzheimer’s Disease, and Parkinson’s Disease. Many single cell technologies have been employed in cancer research in different capacities, such as the use of RNAseq in the study of hetereogeneity of tumors [258], enabling anti-cancer treatment resistance by using single cell detection [259], and the recent scTrio-seq technology introduced by Hou et al. [260]. Another important application in SCT is stem cell research, which opens new frontiers for organ transplantation. SCT in stem cell research has contributed to finding dormant neural cells, which undergo division in accidental situations [261]. To reach the ultimate goal of routine application for clinical settings, two key contests are important. Firstly, investigational costs and time consumption is comparatively high; these need to be abridged to a rational range. Furthermore, it is essential to establish several techniques to test samples, which is repetitive preparation to keep tissue samples in the clinic. This progress will support the clinical application of SCT, which has positive outlooks for cancer diagnostics and treatment for cancer patients.

## 7. Conclusions

In this review article, we have discussed the development of SCT. The entire article gives a broad perspective to the reader about the steps involved in the progress of SCT for diseases analysis. It also deliberates upon the knowledge of current SCT progress and its future trends. The article includes some of the recently developed SCT tools involved in disease diagnosis, analyzing it in single-cell platform and also treating it using novel therapeutic techniques for cargo delivery. The diagnosis mainly highlights the manipulation, detection, sorting, and isolation of single-cells or diseased cells from a bulk of heterogeneous cell types. Lab-on-chip is a well-suited technique that integrates multiple single-cell technologies based on downstream processing requirements. The combined use of microfluidics, laser optics, electrophoretic and electrochemical technology are discussed for single cell diagnosis. Subsequently, we also discussed the devices that are useful for analyzing individual cells to understand their physical and chemical interaction with the microenvironment. In addition, we demonstrated the application of SCT and other tools that have been developed to obtain therapeutics and drug delivery results with high cell viability. This review summarizes the recent trends of single-cell technologies applied for single-cell manipulation, diagnosis, therapeutics, analysis, drug delivery and their future prospects and limitations.

## Figures and Tables

**Table 1:** Single-cell manipulation techniques.

S. No.	Single-Cell Manipulation Techniques Using	Features	Advantages	Disadvantages	References
1	Microfluidics	Droplet microfluidics.	Cell isolation, precise control on small volumes, high throughput screening.	Transportation of droplets is tedious. Special care is required in material choice for functions.	[55,56,57,58]
		Microfluidic DLD, GEDI, GEM.	Efficient cell separation based on size, surface proteins.	Sophisticated fabrication.	[59,60,61]
		Hydrodynamic pressure.	Same chip can be used for multiple process.	Tedious design and fabrication.	[62,63,64]
		Fluidic microarray chip.	High throughput cell manipulation.	Less flexibility in single cell manipulation.	[65,66,67,68]
		Ink jet printing.	Cells can be displaced in the desired pattern.	Small volume of cell culture (8 µL)	[69]
		Micropipette array.	Increased throughput, useful for transporting cells within different microenvironments.	Trapping and release rates are compromised to increase cell viability.	[70]
2	Electric field	Dielectrophoresis.	Useful in handling inside microfluidic channels.	Complex electrode fabrication.	[71,72]
3	Optical energy	Optical tweezers.	Contactless handling, label-free, contamination-free.	Low throughput, high setup cost, substrate-specifi.c	[49,50,51]
		Multiple 3D optical traps.	Increases throughput as compared to optical tweezers, cell detection is incorporated.	Sophisticated instrument setup, diffraction of light limits the maximum number of cells that can be handled at one time.	[73]
4	Acoustic energy	3D acoustic tweezer.	Label-free, contact less, contamination free, safer as compared to the optical technique.	Sophisticated fabrication and calibration process.	[74]

**Table 2:** Single-cell diagnosis techniques.

S. No.	Single-Cell Diagnosis Techniques Using	Features	Advantages	Disadvantages	References
1	Microfluidics	Flow cytometry, A microfluidic technique to sort cells based on their size, granularity, and fluorescence (FACS).	High throughput technique to analyze heterogeneous cell population. Gives high accuracy in sorting.	Not reliable for intracellular diagnosis. Spectral overlap observed in screening of multiple parameters.	[100]
		Cell affinity micro chromatography, a technique based on binding with surface immobilized ligands.	Targeted cells sorted based on surface proteins. Higher sensitivity and selectivity.	Low throughput. Limited by Surface interaction. Complex cell retrieval mechanism.	[101]
		Droplet microfluidics.	Microreactors for individual cells within a droplet, rare cell detection possible.	Huge data analysis required.	[102]
		Micro vortex.	Rare cell isolation is possible. High throughput is possible.	Purity of output is low.	[103]
2	Electric field	Dielectrophoresis technique uses non-uniform electric field.	Useful in the study of neural cells, cancer cells. High efficiency.	Limited to cells showing a response to electric fields. Low throughput.	[104]
3	Optical energy	Photomechanical cell detachment.	single-cell captured per microfluidic chamber allows tracking of individual cells. Surface proteins are preserved. Genetic analysis can provide information about key altering genes in cell lineage.	Low throughput. Expensive setup.	[105]
		Laser capture microdissection.	Allows live single-cell isolation. Even cell organelles can be isolated. Contactless technique.	Requires good optical resolution. Expensive setup.	[106]
5	Magnetic energy	MACS uses functionalized super-paramagnetic nanoparticles to tag cells.	High efficiency. Based on surface markers allows high selectivity and sensitivity. Allows sorting of multiple target cells.	Low throughput. Cell retrieval is difficult.	[107]
6	Omics technology	In Digital droplet, PCR sample is fractionated into droplets using water oil emulsion droplet technology. Each droplet is then allowed to react with the specific primer and probe. The droplets showing positive reaction are quantified.	Very high accuracy and sensitivity in detection of rare DNA target. Quantification of the concentration of targeted cell is possible.	Lengthy process. Redesigning of the process required if results are inconsistent.	[108]

**Table 3:** Single-Cell therapeutic techniques.

S. No.	Single-Cell Therapeutic Techniques Using	Features	Advantages	Disadvantages	References
1	Viral vector	Genetically engineered viruses are used for transfection.	Promising intracellular uptake, targeted delivery.	Immunogenic effects, limitation in genetic material carrying capacity.	[158]
2	Electroporation	Use of electric field for making cell pores.	Cost effective, high delivery efficiency.	Low cell viability, formation of irreversible pore.	[158,165]
3	Optoporation	Use of laser for creating transient cell membrane pores.	Contactless delivery method, high transfection efficiency.	Low throughput for single cell delivery.	[166,167]
4	Mechanoporation	Transient membrane pores are formed by applying mechanical forces on cells.	High cell viability, high transfection efficiency.	Sometimes there is a compromise between high transfection efficiency and high cell viability.	[168,169,170,171]

**Table 4:** Single-cell analysis techniques.

S No.	Single-cell Analysis Techniques	Features	References
1	Single-cell differentiation	4D recording Allows tracking of targeted cell in X, Y and Z directions along with time. Software controlled motorized stage. BaSiC software allows detection of transcription factors as the stem cell undergoes differentiation. W-4PiSMSN allows imaging of cell cross section to view cell organelles based on high-resolution fluorescence microscopy.	[27,195]
2	Single-cell growth	SMR Cell mass and growth rate can be quantified. Quantification based on the resonance of suspended cantilever before and after cell growth cycle. Low-cost technique.	[196]
3	Cell membrane potential measurement	The localized potential of and within a cell can be measured. Useful in the study of neurons.	[197]
4	Intracellular access	This is a live cell extraction technique. Intracellular contents can be extracted over the cell cycle for analysis.	[198]
5	Genomics and Transcriptomics	Study of structure, function, and expression of cell genome/transcriptome (mRNA) in phenotypically varying cells. The transcriptomic study can give connecting link between the genetic makeup and phenotypic characteristics of cells. Uses Whole genome/transcriptome amplification (WGA/WTA) for NGS.	[199]
6	Proteomics	It is the study of cellular protein structure and functions. Uses techniques like mass spectroscopy and antigen-antibody reactions for the study of proteins.	[199]
7	Mass Spectrometry	Useful for analyzing and separating cellular components like DNA, RNA, proteins, metabolites based on their mass. CyTOF useful for detecting single cell surface and intracellular markers.	[200,201]

## References

[1] Jorissen R.N., Walker F., Pouliot N., Garrett T.P.J., Ward C.W., Burgess A.W. (2003). Epidermal growth factor receptor: Mechanisms of activation and signalling. Exp. Cell Res..

[2] Santra T.S., Tseng F.G. (2013). Recent trends on micro/nanofluidic single cell electroporation. Micromachines.

[3] Parsons J.T., Slack-Davis J.K., Tilghman R.W., Iwanicki M., Martin K.H. (2011). Integrin signaling: Cell migration, proliferation, and survival. Handbook of Cell Signaling.

[4] Liu Y., Liu F., Grundke-Iqbal I., Iqbal K., Gong C.-X. (2011). Deficient brain insulin signalling pathway in Alzheimer’s disease and diabetes. J. Pathol..

[5] Zeidán-Chuliá F., de Oliveira B.-H., Salmina A.B., Casanova M.F., Gelain D.P., Noda M., Verkhratsky A., Moreira J.C.F. (2014). Altered expression of Alzheimer’s disease-related genes in the cerebellum of autistic patients: A model for disrupted brain connectome and therapy. Cell Death Dis..

[6] Zuchner T., Schliebs R., Perez-Polo J.R. (2005). Down-regulation of muscarinic acetylcholine receptor M2 adversely affects the expression of Alzheimer’s disease-relevant genes and proteins. J. Neurochem..

[7] Jiang P., Li C., Xiang Z., Jiao B. (2014). Tanshinone IIA reduces the risk of Alzheimer’s disease by inhibiting iNOS, MMP-2 and NF-κBp65 transcription and translation in the temporal lobes of rat models of Alzheimer’s disease. Mol. Med. Rep..

[8] Bhagwat A.S., Vakoc C.R. (2015). Targeting transcription factors in cancer. Trends Cancer.

[9] Wu J.-H., Sun Y.-J., Hsieh P.-H., Shieh G.S. (2013). Inferring coregulation of transcription factors and microRNAs in breast cancer. Gene.

[10] Maulik S., Patel S.D. (1997). Molecular Biotechnology: Therapeutic Applications and Strategies.

[11] Cooper G.M., Ganem D. (1997). The Cell: A Molecular Approach. Nat. Med..

[12] Hamon M., Hong J.W. (2013). New tools and new biology: Recent miniaturized systems for molecular and cellular biology. Mol. Cells.

[13] Swedlow J.R. (2012). Innovation in biological microscopy: Current status and future directions. Bioessays.

[14] Akhtar A., Fuchs E., Mitchison T., Shaw R.J., St Johnston D., Strasser A., Taylor S., Walczak C., Zerial M. (2011). A decade of molecular cell biology: Achievements and challenges. Nat. Rev. Mol. Cell Biol..

[15] Kanapathipillai M., Brock A., Ingber D.E. (2014). Nanoparticle targeting of anti-cancer drugs that alter intracellular signaling or influence the tumor microenvironment. Adv. Drug Deliv. Rev..

[16] Allen T.M., Cullis P.R. (2013). Liposomal drug delivery systems: From concept to clinical applications. Adv. Drug Deliv. Rev..

[17] Agarwal P., Bertozzi C.R. (2015). Site-specific antibody-drug conjugates: The nexus of bioorthogonal chemistry, protein engineering, and drug development. Bioconjug. Chem..

[18] Wen L., Tang F. (2016). Single-cell sequencing in stem cell biology. Genome Biol..

[19] Korthauer K.D., Chu L.-F., Newton M.A., Li Y., Thomson J., Stewart R., Kendziorski C. (2016). A statistical approach for identifying differential distributions in single-cell RNA-seq experiments. Genome Biol..

[20] Kim K.-T., Lee H.W., Lee H.-O., Song H.J., Shin S., Kim H., Shin Y., Nam D.-H., Jeong B.C., Kirsch D.G. (2016). Application of single-cell RNA sequencing in optimizing a combinatorial therapeutic strategy in metastatic renal cell carcinoma. Genome Biol..

[21] Jahn K., Kuipers J., Beerenwinkel N. (2016). Tree inference for single-cell data. Genome Biol..

[22] Heath J.R., Ribas A., Mischel P.S. (2016). Single-cell analysis tools for drug discovery and development. Nat. Rev. Drug Discov..

[23] Neužil P., Campos C.D.M., Wong C.C., Soon J.B.W., Reboud J., Manz A. (2014). From chip-in-a-lab to lab-on-a-chip: Towards a single handheld electronic system for multiple application-specific lab-on-a-chip (ASLOC). Lab Chip.

[24] Whitesides G. (2014). The lab finally comes to the chip!. Lab Chip.

[25] Schulte T.H., Bardell R.L., Weigl B.H. (2002). Microfluidic technologies in clinical diagnostics. Clin. Chim. Acta.

[26] Tseng F.-G., Santra T.S. (2014). Micro/Nano Fluidic Devices for Single Cell Analysis. Micromachines.

[27] Tseng F.-G., Santra T.S. (2016). Essentials of Single-Cell Analysis: Concepts, Applications and Future Prospects. Essentials of Single-Cell Analysis: Concepts, Applications and Future Prospects.

[28] Chattopadhyay P.K., Gierahn T.M., Roederer M., Love J.C. (2014). Single-cell technologies for monitoring immune systems. Nat. Immunol..

[29] Liang S.-B., Fu L.-W. (2017). Application of single-cell technology in cancer research. Biotechnol. Adv..

[30] Fritzsch F.S.O., Dusny C., Frick O., Schmid A. (2012). Single-cell analysis in biotechnology, systems biology, and biocatalysis. Annu. Rev. Chem. Biomol. Eng..

[31] Tseng F.-G., Santra T.S. (2016). Single Cell Analysis in Biotechnology and System Biology.

[32] Kar S., Mohan L., Dey K., Shinde P., Chang H.-Y., Nagai M., Santra T.S. (2018). Single Cell Electroporation-Current Trends, Applications and Future prospects. J. Micromech. Microeng..

[33] Kumar A., Mohan L., Shinde P., Chang H.Y., Nagai M., Santra T.S., Santra T., Tseng F.G. (2018). Mechanoporation: Toward Single Cell Approaches. Handbook of Single Cell Technologies.

[34] Skelley A.M., Kirak O., Suh H., Jaenisch R., Voldman J. (2009). Microfluidic control of cell pairing and fusion. Nat. Methods.

[35] Frimat J.-P., Becker M., Chiang Y.-Y., Marggraf U., Janasek D., Hengstler J.G., Franzke J., West J. (2011). A microfluidic array with cellular valving for single cell co-culture. Lab Chip.

[36] Hong S., Pan Q., Lee L.P. (2012). Single-cell level co-culture platform for intercellular communication. Integr. Biol..

[37] Chen Y.-C., Cheng Y.-H., Kim H.S., Ingram P.N., Nor J.E., Yoon E. (2014). Paired single cell co-culture microenvironments isolated by two-phase flow with continuous nutrient renewal. Lab Chip.

[38] Huang W.-H., Cheng W., Zhang Z., Pang D.-W., Wang Z.-L., Cheng J.-K., Cui D.-F. (2004). Transport, location, and quantal release monitoring of single cells on a microfluidic device. Anal. Chem..

[39] Thielecke H., Stieglitz T., Beutel H., Matthies T., Ruf H.H., Meyer J.-U. (1999). Fast and precise positioning of single cells on planar electrode substrates. IEEE Eng. Med. Biol. Mag..

[40] Wheeler A.R., Throndset W.R., Whelan R.J., Leach A.M., Zare R.N., Liao Y.H., Farrell K., Manger I.D., Daridon A. (2003). Microfluidic device for single-cell analysis. Anal. Chem..

[41] Yun K.-S., Yoon E. (2005). Micro/nanofluidic device for single-cell-based assay. Biomed. Microdevices.

[42] Müller T., Gradl G., Howitz S., Shirley S., Schnelle T., Fuhr G. (1999). A 3-D microelectrode system for handling and caging single cells and particles. Biosens. Bioelectron..

[43] Taff B.M., Voldman J. (2005). A scalable addressable positive-dielectrophoretic cell-sorting array. Anal. Chem..

[44] Manaresi N., Romani A., Medoro G., Altomare L., Leonardi A., Tartagni M., Guerrieri R. (2003). A CMOS chip for individual cell manipulation and detection. IEEE J. Solid-State Circuits.

[45] Park H., Kim D., Yun K.S. (2010). Single-cell manipulation on microfluidic chip by dielectrophoretic actuation and impedance detection. Sens. Actuators B Chem..

[46] Gray D.S., Tan J.L., Voldman J., Chen C.S. (2004). Dielectrophoretic registration of living cells to a microelectrode array. Biosens. Bioelectron..

[47] Albrecht D.R., Tsang V.L., Sah R.L., Bhatia S.N. (2005). Photo-and electropatterning of hydrogel-encapsulated living cell arrays. Lab Chip.

[48] Toriello N.M., Douglas E.S., Mathies R.A. (2005). Microfluidic device for electric field-driven single-cell capture and activation. Anal. Chem..

[49] Juan M.L., Righini M., Quidant R. (2011). Plasmon nano-optical tweezers. Nat. Photonics.

[50] Chiou P.Y., Ohta A.T., Wu M.C. (2005). Massively parallel manipulation of single cells and microparticles using optical images. Nature.

[51] Mirsaidov U., Scrimgeour J., Timp W., Beck K., Mir M., Matsudaira P., Timp G. (2008). Live cell lithography: Using optical tweezers to create synthetic tissue. Lab Chip.

[52] Ashkin A., Dziedzic J.M., Yamane T. (1987). Optical trapping and manipulation of single cells using infrared laser beams. Nature.

[53] Arai F., Ichikawa A., Ogawa M., Fukuda T., Horio K., Itoigawa K. (2001). High-speed separation system of randomly suspended single living cells by laser trap and dielectrophoresis. Electrophoresis.

[54] Schroder B.W., Johnson B.M., Garrity D.M., Dasi L.P., Krapf D. (2014). Force spectroscopy in the bloodstream of live embryonic zebrafish with optical tweezers. Frontiers in Optics.

[55] Theberge A.B., Courtois F., Schaerli Y., Fischlechner M., Abell C., Hollfelder F., Huck W.T.S. (2010). Microdroplets in microfluidics: An evolving platform for discoveries in chemistry and biology. Angew. Chem. Int. Ed..

[56] Shang L., Cheng Y., Zhao Y. (2017). Emerging droplet microfluidics. Chem. Rev..

[57] Clausell-Tormos J., Lieber D., Baret J.-C., El-Harrak A., Miller O.J., Frenz L., Blouwolff J., Humphry K.J., Köster S., Duan H. (2008). Droplet-based microfluidic platforms for the encapsulation and screening of mammalian cells and multicellular organisms. Chem. Biol..

[58] Sukhatme S., Agarwal A. (2012). Digital microfluidics: Techniques, their applications and advantages. J. Bioeng. Biomed. Sci..

[59] Karabacak N.M., Spuhler P.S., Fachin F., Lim E.J., Pai V., Ozkumur E., Martel J.M., Kojic N., Smith K., Chen P. (2014). Microfluidic, marker-free isolation of circulating tumor cells from blood samples. Nat. Protoc..

[60] Kirby B.J., Jodari M., Loftus M.S., Gakhar G., Pratt E.D., Chanel-Vos C., Gleghorn J.P., Santana S.M., Liu H., Smith J.P. (2012). Functional characterization of circulating tumor cells with a prostate-cancer-specific microfluidic device. PLoS ONE.

[61] Sheng W., Ogunwobi O.O., Chen T., Zhang J., George T.J., Liu C., Fan Z.H. (2014). Capture, release and culture of circulating tumor cells from pancreatic cancer patients using an enhanced mixing chip. Lab Chip.

[62] Arakawa T., Noguchi M., Sumitomo K., Yamaguchi Y., Shoji S. (2011). High-throughput single-cell manipulation system for a large number of target cells. Biomicrofluidics.

[63] Bragheri F., Osellame R. (2017). Hydrodynamic lift for single cell manipulation in a femtosecond laser fabricated optofluidic chip. Optofluidics Microfluidics Nanofluidics.

[64] Lincoln B., Schinkinger S., Travis K., Wottawah F., Ebert S., Sauer F., Guck J. (2007). Reconfigurable microfluidic integration of a dual-beam laser trap with biomedical applications. Biomed. Microdevices.

[65] Di Carlo D., Wu L.Y., Lee L.P. (2006). Dynamic single cell culture array. Lab Chip.

[66] Wlodkowic D., Faley S., Zagnoni M., Wikswo J.P., Cooper J.M. (2009). Microfluidic single-cell array cytometry for the analysis of tumor apoptosis. Anal. Chem..

[67] Faley S.L., Copland M., Wlodkowic D., Kolch W., Seale K.T., Wikswo J.P., Cooper J.M. (2009). Microfluidic single cell arrays to interrogate signalling dynamics of individual, patient-derived hematopoietic stem cells. Lab Chip.

[68] Xu X., Sarder P., Li Z., Nehorai A. (2013). Optimization of microfluidic microsphere-trap arrays. Biomicrofluidics.

[69] Yusof A., Keegan H., Spillane C.D., Sheils O.M., Martin C.M., O’Leary J.J., Zengerle R., Koltay P. (2011). Inkjet-like printing of single-cells. Lab Chip.

[70] Nagai M., Oohara K., Kato K., Kawashima T., Shibata T. (2015). Development and characterization of hollow microprobe array as a potential tool for versatile and massively parallel manipulation of single cells. Biomed. Microdevices.

[71] Dastani K., Moghimi Zand M., Hadi A. (2017). Dielectrophoretic effect of nonuniform electric fields on the protoplast cell. J. Comput. Appl. Mech..

[72] Fernádez-Morales F.H., Duarte J.E., Samitier-Mart’i J. (2008). Bacterial handling under the influence of non-uniform electric fields: Dielectrophoretic and electrohydrodynamic effects. An. Acad. Bras. Cienc..

[73] Wang X., Yan X., Chen S., Sun D. Automated parallel cell isolation and deposition using microwell array and optical tweezers. Proceedings of the 2012 IEEE International Conference on Robotics and Automation (ICRA).

[74] Guo F., Mao Z., Chen Y., Xie Z., Lata J.P., Li P., Ren L., Liu J., Yang J., Dao M. (2016). Three-dimensional manipulation of single cells using surface acoustic waves. Proc. Natl. Acad. Sci. USA.

[75] Brouzes E., Medkova M., Savenelli N., Marran D., Twardowski M., Hutchison J.B., Rothberg J.M., Link D.R., Perrimon N., Samuels M.L. (2009). Droplet microfluidic technology for single-cell high-throughput screening. Proc. Natl. Acad. Sci. USA.

[76] Zhu Z., Yang C.J. (2016). Hydrogel droplet microfluidics for high-throughput single molecule/cell analysis. Acc. Chem. Res..

[77] Mandrycky C., Wang Z., Kim K., Kim D.-H. (2016). 3D bioprinting for engineering complex tissues. Biotechnol. Adv..

[78] Zheng S., Lin H.K., Lu B., Williams A., Datar R., Cote R.J., Tai Y.-C. (2011). 3D microfilter device for viable circulating tumor cell (CTC) enrichment from blood. Biomed. Microdevices.

[79] Tang Y., Shi J., Li S., Wang L., Cayre Y.E., Chen Y. (2014). Microfluidic device with integrated microfilter of conical-shaped holes for high efficiency and high purity capture of circulating tumor cells. Sci. Rep..

[80] Nagrath S., Sequist L.V., Maheswaran S., Bell D.W., Irimia D., Ulkus L., Smith M.R., Kwak E.L., Digumarthy S., Muzikansky A. (2007). Isolation of rare circulating tumour cells in cancer patients by microchip technology. Nature.

[81] Biran I., Walt D.R. (2002). Optical imaging fiber-based single live cell arrays: A high-density cell assay platform. Anal. Chem..

[82] Revzin A., Sekine K., Sin A., Tompkins R.G., Toner M. (2005). Development of a microfabricated cytometry platform for characterization and sorting of individual leukocytes. Lab Chip.

[83] Yun K.-S., Yoon E. A micro/nano-fluidic chip-based micro-well array for high-throughput cell analysis and drug screening. Proceedings of the International Conference on Miniaturized Chemical and Blochemlcal Analysts Systems.

[84] Wilson C.F., Simpson G.J., Chiu D.T., Strömberg A., Orwar O., Rodriguez N., Zare R.N. (2001). Nanoengineered structures for holding and manipulating liposomes and cells. Anal. Chem..

[85] Sarder P., Nehorai A. (2011). Statistical design of position-encoded microsphere arrays. IEEE Trans. Nanobiosci..

[86] Hunt T.P., Westervelt R.M. (2006). Dielectrophoresis tweezers for single cell manipulation. Biomed. Microdevices.

[87] Kodama T., Osaki T., Kawano R., Kamiya K., Miki N., Takeuchi S. (2013). Round-tip dielectrophoresis-based tweezers for single micro-object manipulation. Biosens. Bioelectron..

[88] Park I.S., Eom K., Son J., Chang W.-J., Park K., Kwon T., Yoon D.S., Bashir R., Lee S.W. (2012). Microfluidic multifunctional probe array dielectrophoretic force spectroscopy with wide loading rates. ACS Nano.

[89] Kim M.H., Lee J., Nam K., Park I.S., Son M., Ko H., Lee S., Yoon D.S., Chang W.-J., Lee S.Y. (2017). Automated Dielectrophoretic Tweezers-Based Force Spectroscopy System in a Microfluidic Device. Sensors.

[90] Chuang H.-S., Ku H.-Y., Li F.-T., Kumar A., Wang J.-C., Wang K.-C. (2016). Optoelectrokinetic Manipulation for Cell Analysis. Essentials of Single-Cell Analysis.

[91] Casey D., Dooley J. (2016). Optical tools for single-cell manipulation and analysis. Essentials of Single-Cell Analysis.

[92] Liberale C., Cojoc G., Bragheri F., Minzioni P., Perozziello G., La Rocca R., Ferrara L., Rajamanickam V., Di Fabrizio E., Cristiani I. (2013). Integrated microfluidic device for single-cell trapping and spectroscopy. Sci. Rep..

[93] Wang X., Gou X., Chen S., Yan X., Sun D. (2013). Cell manipulation tool with combined microwell array and optical tweezers for cell isolation and deposition. J. Micromech. Microeng..

[94] Wang X., Chen S., Kong M., Wang Z., Costa K.D., Li R.A., Sun D. (2011). Enhanced cell sorting and manipulation with combined optical tweezer and microfluidic chip technologies. Lab Chip.

[95] Ding X., Lin S.-C.S., Kiraly B., Yue H., Li S., Chiang I.-K., Shi J., Benkovic S.J., Huang T.J. (2012). On-chip manipulation of single microparticles, cells, and organisms using surface acoustic waves. Proc. Natl. Acad. Sci. USA.

[96] Fulwyler M.J. (1974). Status quo in flow-through cytometry. J. Histochem. Cytochem..

[97] Wilkerson M.J. (2012). Principles and applications of flow cytometry and cell sorting in companion animal medicine. Vet. Clin. Small Anim. Pract..

[98] Reggeti F., Bienzle D. (2011). Flow cytometry in veterinary oncology. Vet. Pathol..

[99] Adan A., Alizada G., Kiraz Y., Baran Y., Nalbant A. (2017). Flow cytometry: Basic principles and applications. Crit. Rev. Biotechnol..

[100] Hu P., Zhang W., Xin H., Deng G. (2016). Single cell isolation and analysis. Front. Cell Dev. Biol..

[101] Herling T.W., O’Connell D.J., Bauer M.C., Persson J., Weininger U., Knowles T.P.J., Linse S. (2016). A microfluidic platform for real-time detection and quantification of protein-ligand interactions. Biophys. J..

[102] Rajan S., Kierny M.R., Mercer A., Wu J., Tovchigrechko A., Wu H., Dall W.F., Xiao X., Chowdhury P.S. (2018). Recombinant human B cell repertoires enable screening for rare, specific, and natively paired antibodies. Commun. Biol..

[103] Khojah R., Stoutamore R., Di Carlo D. (2017). Size-tunable microvortex capture of rare cells. Lab Chip.

[104] Chen J., Li J., Sun Y. (2012). Microfluidic approaches for cancer cell detection, characterization, and separation. Lab Chip.

[105] Chen Y.C., Baac H.W., Lee K.T., Fouladdel S., Teichert K., Ok J.G., Cheng Y.H., Ingram P.N., Hart A.J., Azizi E. (2017). Selective Photomechanical Detachment and Retrieval of Divided Sister Cells from Enclosed Microfluidics for Downstream Analyses. ACS Nano.

[106] Chung S.H., Shen W. (2015). Laser capture microdissection: From its principle to applications in research on neurodegeneration. Neural Regen. Res..

[107] Adams J.D., Kim U., Soh H.T. (2008). Multitarget magnetic activated cell sorter. Proc. Natl. Acad. Sci. USA.

[108] Taylor S.C., Laperriere G., Germain H. (2017). Droplet Digital PCR versus qPCR for gene expression analysis with low abundant targets: From variable nonsense to publication quality data. Sci. Rep..

[109] Piyasena M.E., Graves S.W. (2014). The intersection of flow cytometry with microfluidics and microfabrication. Lab Chip.

[110] Croker A.K., Goodale D., Chu J., Postenka C., Hedley B.D., Hess D.A., Allan A.L. (2009). High aldehyde dehydrogenase and expression of cancer stem cell markers selects for breast cancer cells with enhanced malignant and metastatic ability. J. Cell. Mol. Med..

[111] Prestegarden L., Svendsen A., Wang J., Sleire L., Skaftnesmo K.O., Bjerkvig R., Yan T., Askland L., Persson A., Sakariassen P.Ø. (2010). Glioma cell populations grouped by different cell type markers drive brain tumor growth. Cancer Res..

[112] Gracz A.D., Williamson I.A., Roche K.C., Johnston M.J., Wang F., Wang Y., Attayek P.J., Balowski J., Liu X.F., Laurenza R.J. (2015). A high-throughput platform for stem cell niche co-cultures and downstream gene expression analysis. Nat. Cell Biol..

[113] Mahdieh N., Rabbani B. (2013). An overview of mutation detection methods in genetic disorders. Iran. J. Pediatr..

[114] Hung P.J., Lee P.J., Sabounchi P., Lin R., Lee L.P. (2005). Continuous perfusion microfluidic cell culture array for high-throughput cell-based assays. Biotechnol. Bioeng..

[115] Cheng Y.-H., Chen Y.-C., Brien R., Yoon E. (2016). Scaling and automation of a high-throughput single-cell-derived tumor sphere assay chip. Lab Chip.

[116] Whitesides G.M. (2006). The origins and the future of microfluidics. Nature.

[117] Chen Y.-C., Ingram P.N., Fouladdel S., McDermott S.P., Azizi E., Wicha M.S., Yoon E. (2016). High-Throughput Single-Cell Derived Sphere Formation for Cancer Stem-Like Cell Identification and Analysis. Sci. Rep..

[118] Lecault V., VanInsberghe M., Sekulovic S., Knapp D.J.H.F., Wohrer S., Bowden W., Viel F., McLaughlin T., Jarandehei A., Miller M. (2011). High-throughput analysis of single hematopoietic stem cell proliferation in microfluidic cell culture arrays. Nat. Methods.

[119] Zhang Z., Chen Y.-C., Cheng Y.-H., Luan Y., Yoon E. (2016). Microfluidics 3D gel-island chip for single cell isolation and lineage-dependent drug responses study. Lab Chip.

[120] Choi Y.-J., Ingram P.N., Yang K., Coffman L., Iyengar M., Bai S., Thomas D.G., Yoon E., Buckanovich R.J. (2015). Identifying an ovarian cancer cell hierarchy regulated by bone morphogenetic protein 2. Proc. Natl. Acad. Sci. USA.

[121] Reya T., Morrison S.J., Clarke M.F., Weissman I.L. (2001). Stem cells, cancer, and cancer stem cells. Nature.

[122] Canavan H.E., Cheng X., Graham D.J., Ratner B.D., Castner D.G. (2005). Cell sheet detachment affects the extracellular matrix: A surface science study comparing thermal liftoff, enzymatic, and mechanical methods. J. Biomed. Mater. Res. Part A.

[123] Sumaru K., Kikuchi K., Takagi T., Yamaguchi M., Satoh T., Morishita K., Kanamori T. (2013). On-demand killing of adherent cells on photo-acid-generating culture substrates. Biotechnol. Bioeng..

[124] Guillaume-Gentil O., Zambelli T., Vorholt J.A. (2014). Isolation of single mammalian cells from adherent cultures by fluidic force microscopy. Lab Chip.

[125] Kudo L.C., Vi N., Ma Z., Fields T., Avliyakulov N.K., Haykinson M.J., Bragin A., Karsten S.L. (2012). Novel cell and tissue acquisition system (CTAS): Microdissection of live and frozen brain tissues. PLoS ONE.

[126] Sada T., Fujigaya T., Niidome Y., Nakazawa K., Nakashima N. (2011). Near-IR laser-triggered target cell collection using a carbon nanotube-based cell-cultured substrate. ACS Nano.

[127] Baac H.W., Lee T., Guo L.J. (2013). Micro-ultrasonic cleaving of cell clusters by laser-generated focused ultrasound and its mechanisms. Biomed. Opt. Express.

[128] Baac H.W., Ok J.G., Maxwell A., Lee K.-T., Chen Y.-C., Hart A.J., Xu Z., Yoon E., Guo L.J. (2012). Carbon-nanotube optoacoustic lens for focused ultrasound generation and high-precision targeted therapy. Sci. Rep..

[129] Beaudet A.L. (2016). Using fetal cells for prenatal diagnosis: History and recent progress. Am. J. Med. Genet. Part C.

[130] Emad A., Bouchard E.F., Lamoureux J., Ouellet A., Dutta A., Klingbeil U., Drouin R. (2014). Validation of automatic scanning of microscope slides in recovering rare cellular events: Application for detection of fetal cells in maternal blood. Prenat. Diagn..

[131] Breman A.M., Chow J.C., U’ren L., Normand E.A., Qdaisat S., Zhao L., Henke D.M., Chen R., Shaw C.A., Jackson L. (2016). Evidence for feasibility of fetal trophoblastic cell-based noninvasive prenatal testing. Prenat. Diagn..

[132] Mouawia H., Saker A., Jais J.-P., Benachi A., Bussières L., Lacour B., Bonnefont J.-P., Frydman R., Simpson J.L., Paterlini-Brechot P. (2012). Circulating trophoblastic cells provide genetic diagnosis in 63 fetuses at risk for cystic fibrosis or spinal muscular atrophy. Reprod. Biomed. Online.

[133] Kølvraa S., Singh R., Normand E.A., Qdaisat S., Veyver I.B., Jackson L., Hatt L., Schelde P., Uldbjerg N., Vestergaard E.M. (2016). Genome-wide copy number analysis on DNA from fetal cells isolated from the blood of pregnant women. Prenat. Diagn..

[134] Mavrou A., Kouvidi E., Antsaklis A., Souka A., Kitsiou Tzeli S., Kolialexi A. (2007). Identification of nucleated red blood cells in maternal circulation: A second step in screening for fetal aneuploidies and pregnancy complications. Prenat. Diagn..

[135] Kwon K.H., Jeon Y.J., Hwang H.S., Lee K.A., Kim Y.J., Chung H.W., Pang M.G. (2007). A high yield of fetal nucleated red blood cells isolated using optimal osmolality and a double-density gradient system. Prenat. Diagn..

[136] Bhat N.M., Bieber M.M., Teng N.N.H. (1993). One-step enrichment of nucleated red blood cells: A potential application in perinatal diagnosis. J. Immunol. Methods.

[137] De Wit H., Nabbe K.C.A.M., Kooren J.A., Adriaansen H.J., Roelandse-Koop E.A., Schuitemaker J.H.N., Hoffmann J.J.M.L. (2011). Reference values of fetal erythrocytes in maternal blood during pregnancy established using flow cytometry. Am. J. Clin. Pathol..

[138] Herzenberg L.A., Bianchi D.W., Schröder J., Cann H.M., Iverson G.M. (1979). Fetal cells in the blood of pregnant women: Detection and enrichment by fluorescence-activated cell sorting. Proc. Natl. Acad. Sci. USA.

[139] He Z., Guo F., Feng C., Cai B., Lata J.P., He R., Huang Q., Yu X., Rao L., Liu H. (2017). Fetal nucleated red blood cell analysis for non-invasive prenatal diagnostics using a nanostructure microchip. J. Mater. Chem. B.

[140] Kantak C., Chang C.-P., Wong C.C., Mahyuddin A., Choolani M., Rahman A. (2014). Lab-on-a-chip technology: Impacting non-invasive prenatal diagnostics (NIPD) through miniaturisation. Lab Chip.

[141] Chen J.-F., Zhu Y., Lu Y.-T., Hodara E., Hou S., Agopian V.G., Tomlinson J.S., Posadas E.M., Tseng H.-R. (2016). Clinical applications of NanoVelcro rare-cell assays for detection and characterization of circulating tumor cells. Theranostics.

[142] Lin M., Chen J.-F., Lu Y.-T., Zhang Y., Song J., Hou S., Ke Z., Tseng H.-R. (2014). Nanostructure embedded microchips for detection, isolation, and characterization of circulating tumor cells. Acc. Chem. Res..

[143] Ke Z., Lin M., Chen J.-F., Choi J., Zhang Y., Fong A., Liang A.-J., Chen S.-F., Li Q., Fang W. (2014). Programming thermoresponsiveness of NanoVelcro substrates enables effective purification of circulating tumor cells in lung cancer patients. ACS Nano.

[144] Hou M., Zhang Y., Chen J.-F., Lin M., Wang L., Hou S., Tseng H.-R., Kuang M., Ke Z. (2016). Detecting ALK-rearrangement of CTC enriched by nanovelcro chip in advanced NSCLC patients. Oncotarget.

[145] Zhao L., Tang C., Xu L., Zhang Z., Li X., Hu H., Cheng S., Zhou W., Huang M., Fong A. (2016). Enhanced and differential capture of circulating tumor cells from lung cancer patients by microfluidic assays using aptamer cocktail. Small.

[146] Liu S., Tian Z., Zhang L., Hou S., Hu S., Wu J., Jing Y., Sun H., Yu F., Zhao L. (2016). Combined cell surface carbonic anhydrase 9 and CD147 antigens enable high-efficiency capture of circulating tumor cells in clear cell renal cell carcinoma patients. Oncotarget.

[147] Ankeny J.S., Hou S., Li Q., Song M., Wu D., Chen J.F., Lee T., Lin M., Sho S., Rochefort M.M. (2016). Circulating tumour cells as a biomarker for diagnosis and staging in pancreatic cancer. Br. J. Cancer.

[148] Wang S., Liu K., Liu J., Yu Z.T.-F., Xu X., Zhao L., Lee T., Lee E.K., Reiss J., Lee Y.-K. (2011). Highly efficient capture of circulating tumor cells by using nanostructured silicon substrates with integrated chaotic micromixers. Angew. Chem. Int. Ed..

[149] Chen J.-F., Ho H., Lichterman J., Lu Y.-T., Zhang Y., Garcia M.A., Chen S.-F., Liang A.-J., Hodara E., Zhau H.E. (2015). Subclassification of prostate cancer circulating tumor cells by nuclear size reveals very small nuclear circulating tumor cells in patients with visceral metastases. Cancer.

[150] Lu Y.-T., Zhao L., Shen Q., Garcia M.A., Wu D., Hou S., Song M., Xu X., OuYang W.-H., OuYang W.W.-L. (2013). NanoVelcro Chip for CTC enumeration in prostate cancer patients. Methods.

[151] Hou S., Chen J.-F., Song M., Zhu Y., Jan Y.J., Chen S.H., Weng T.-H., Ling D.-A., Chen S.-F., Ro T. (2017). Imprinted NanoVelcro Microchips for Isolation and Characterization of Circulating Fetal Trophoblasts: Toward Noninvasive Prenatal Diagnostics. ACS Nano.

[152] Yamamura S., Kishi H., Tokimitsu Y., Kondo S., Honda R., Rao S.R., Omori M., Tamiya E., Muraguchi A. (2005). Single-cell microarray for analyzing cellular response. Anal. Chem..

[153] Song Q., Han Q., Bradshaw E.M., Kent S.C., Raddassi K., Nilsson B., Nepom G.T., Hafler D.A., Love J.C. (2009). On-chip activation and subsequent detection of individual antigen-specific T cells. Anal. Chem..

[154] Jin A., Ozawa T., Tajiri K., Obata T., Kondo S., Kinoshita K., Kadowaki S., Takahashi K., Sugiyama T., Kishi H. (2009). A rapid and efficient single-cell manipulation method for screening antigen-specific antibody-secreting cells from human peripheral blood. Nat. Med..

[155] Stott S.L., Hsu C.-H., Tsukrov D.I., Yu M., Miyamoto D.T., Waltman B.A., Rothenberg S.M., Shah A.M., Smas M.E., Korir G.K. (2010). Isolation of circulating tumor cells using a microvortex-generating herringbone-chip. Proc. Natl. Acad. Sci. USA.

[156] Stroock A.D., Dertinger S.K.W., Ajdari A., Mezić I., Stone H.A., Whitesides G.M. (2002). Chaotic mixer for microchannels. Science.

[157] Leslie M. (2011). The power of one. Science.

[158] Yin L., Fu S.Q., Nanakorn T., Garcia-Sanchez F., Chung I., Pizzorno G., Hanania E., Deisseroth A., Cote R., Heimfeld S. (1998). Results of retroviral and adenoviral approaches to cancer gene therapy. Stem Cells.

[159] Schmid R.M., Weidenbach H., Draenert G.F., Liptay S., Lührs H., Adler G. (1997). Liposome mediated gene transfer into the rat oesophagus. Gut.

[160] Capecchi M.R. (1980). High efficiency transformation by direct microinjection of DNA into cultured mammalian cells. Cell.

[161] Ohta S., Suzuki K., Ogino Y., Miyagawa S., Murashima A., Matsumaru D., Yamada G. (2008). Gene transduction by sonoporation. Dev. Growth Differ..

[162] Santra T.S., Wang P.-C., Chang H.-Y., Tseng F.-G. (2013). Tuning nano electric field to affect restrictive membrane area on localized single cell nano-electroporation. Appl. Phys. Lett..

[163] Santra T.S., Chang H.-Y., Wang P.-C., Tseng F.-G. (2014). Impact of pulse duration on localized single-cell nano-electroporation. Analyst.

[164] Takahata K. (2013). Advances in Micro/Nano Electromechanical Systems and Fabrication Technologies.

[165] Longo P.A., Kavran J.M., Kim M.-S., Leahy D.J. (2013). Generating mammalian stable cell lines by electroporation. Methods in Enzymology.

[166] Waleed M., Hwang S.-U., Kim J.-D., Shabbir I., Shin S.-M., Lee Y.-G. (2013). Single-cell optoporation and transfection using femtosecond laser and optical tweezers. Biomed. Opt. Express.

[167] Wu T.-H., Teslaa T., Kalim S., French C.T., Moghadam S., Wall R., Miller J.F., Witte O.N., Teitell M.A., Chiou P.-Y. (2011). Photothermal nanoblade for large cargo delivery into mammalian cells. Anal. Chem..

[168] Chun K., Hashiguchi G., Hiroyuki Fujita H.T. (1999). Fabrication of array of hollow microcapillaries used for injection of genetic materials into animal/plant cells. Jpn. J. Appl. Phys..

[169] Guenat O.T., Generelli S., Dadras M., Berdondini L., de Rooij N.F., Koudelka-Hep M. (2005). Generic technological platform for microfabricating silicon nitride micro-and nanopipette arrays. J. Micromech. Microeng..

[170] Sharei A., Zoldan J., Adamo A., Sim W.Y., Cho N., Jackson E., Mao S., Schneider S., Han M.-J., Lytton-Jean A. (2013). A vector-free microfluidic platform for intracellular delivery. Proc. Natl. Acad. Sci. USA.

[171] Lee Szeto G., Van Egeren D., Worku H., Sharei A., Alejandro B., Park C., Frew K., Brefo M., Mao S., Heimann M. (2015). Microfluidic squeezing for intracellular antigen loading in polyclonal B-cells as cellular vaccines. Sci. Rep..

[172] Teissie J., Golzio M., Rols M.P. (2005). Mechanisms of cell membrane electropermeabilization: A minireview of our present (lack of?) knowledge. Biochim. Biophys. Acta Gen. Subj..

[173] Lundqvist J.A., Sahlin F., Åberg M.A.I., Strömberg A., Eriksson P.S., Orwar O. (1998). Altering the biochemical state of individual cultured cells and organelles with ultramicroelectrodes. Proc. Natl. Acad. Sci. USA.

[174] Haas K., Sin W.-C., Javaherian A., Li Z., Cline H.T. (2001). Single-cell electroporationfor gene transfer in vivo. Neuron.

[175] Huang Y., Rubinsky B. (2001). Microfabricated electroporation chip for single cell membrane permeabilization. Sens. Actuators A Phys..

[176] Khine M., Lau A., Ionescu-Zanetti C., Seo J., Lee L.P. (2005). A single cell electroporation chip. Lab Chip.

[177] Potter H., Heller R. (2018). Transfection by electroporation. Curr. Protoc. Mol. Biol..

[178] Prasanna G.L., Panda T. (1997). Electroporation: Basic principles, practical considerations and applications in molecular biology. Bioprocess Biosyst. Eng..

[179] Santra T.S., Chen C.-W., Chang H.-Y., Tseng F.-G. (2016). Dielectric passivation layer as a substratum on localized single-cell electroporation. Rsc Adv..

[180] Kang W., Yavari F., Minary-Jolandan M., Giraldo-Vela J.P., Safi A., McNaughton R.L., Parpoil V., Espinosa H.D. (2013). Nanofountain probe electroporation (NFP-E) of single cells. Nano Lett..

[181] Santra T.S., Kar S., Borana J., Wang P.-C., Tseng F.-G. (2014). Nanolocalized Single-Cell-Membrane Nanoelectroporation: For higher efficiency with high cell viability. IEEE Nanotechnol. Mag..

[182] Jiang H. (2016). Optical MEMS for Chemical Analysis and Biomedicine.

[183] Xiong R., Samal S.K., Demeester J., Skirtach A.G., De Smedt S.C., Braeckmans K. (2016). Laser-assisted photoporation: Fundamentals, technological advances and applications. Adv. Phys. X.

[184] Tsukakoshi M., Kurata S., Nomiya Y., Ikawa Y., Kasuya T. (1984). A novel method of DNA transfection by laser microbeam cell surgery. Appl. Phys. B Lasers Opt..

[185] Nikolskaya A.V., Nikolski V.P., Efimov I.R. (2013). Transfection of Cardiac Cells by Means of Laser-Assisted Optoporation. Handbook of Biophotonics.

[186] Davis A.A., Farrar M.J., Nishimura N., Jin M.M., Schaffer C.B. (2013). Optoporation and genetic manipulation of cells using femtosecond laser pulses. Biophys. J..

[187] Lalonde B.S.-L., Boulais É., Lebrun J.-J., Meunier M. (2013). Visible and near infrared resonance plasmonic enhanced nanosecond laser optoporation of cancer cells. Biomed. Opt. Express.

[188] Xiong R., Raemdonck K., Peynshaert K., Lentacker I., De Cock I., Demeester J., De Smedt S.C., Skirtach A.G., Braeckmans K. (2014). Comparison of gold nanoparticle mediated photoporation: Vapor nanobubbles outperform direct heating for delivering macromolecules in live cells. ACS Nano.

[189] Lakshmanan S., Gupta G.K., Avci P., Chandran R., Sadasivam M., Jorge A.E.S., Hamblin M.R. (2014). Physical energy for drug delivery; poration, concentration and activation. Adv. Drug Deliv. Rev..

[190] Wu T.-H., Teslaa T., Teitell M.A., Chiou P.-Y. (2010). Photothermal nanoblade for patterned cell membrane cutting. Opt. Express.

[191] Peer E., Artzy-Schnirman A., Gepstein L., Sivan U. (2012). Hollow nanoneedle array and its utilization for repeated administration of biomolecules to the same cells. ACS Nano.

[192] Park S., Kim Y.-S., Kim W.B., Jon S. (2009). Carbon nanosyringe array as a platform for intracellular delivery. Nano Lett..

[193] Shibata T., Yamanaka S., Kato N., Kawashima T., Nomura M., Mineta T., Makino E. (2009). Fabrication of micromanipulator array for cell patterning. Microelectron. Eng..

[194] Lai D., Labuz J.M., Kim J., Luker G.D., Shikanov A., Takayama S. (2013). Simple multi-level microchannel fabrication by pseudo-grayscale backside diffused light lithography. RSC Adv..

[195] Peng T., Thorn K., Schroeder T., Wang L., Theis F.J., Marr C., Navab N. (2017). A BaSiC tool for background and shading correction of optical microscopy images. Nat. Commun..

[196] Cermak N., Olcum S., Delgado F.F., Wasserman S.C., Payer K.R., Murakami M.A., Knudsen S.M., Kimmerling R.J., Stevens M.M., Kikuchi Y. (2016). High-throughput measurement of single-cell growth rates using serial microfluidic mass sensor arrays. Nat. Biotechnol..

[197] Liu R., Chen R., Elthakeb A.T., Lee S.H., Hinckley S., Khraiche M.L., Scott J., Pre D., Hwang Y., Tanaka A. (2017). High Density Individually Addressable Nanowire Arrays Record Intracellular Activity from Primary Rodent and Human Stem Cell Derived Neurons. Nano Lett..

[198] Cao Y., Hjort M., Chen H., Birey F., Leal-Ortiz S.A., Han C.M., Santiago J.G., Pacsca S.P., Wu J.C., Melosh N.A. (2017). Nondestructive nanostraw intracellular sampling for longitudinal cell monitoring. Proc. Natl. Acad. Sci. USA.

[199] Horgan R.P., Kenny L.C. (2011). ‘Omic’ technologies: Genomics, transcriptomics, proteomics and metabolomics. Obstet. Gynaecol..

[200] Li L., Garden R.W., Sweedler J. (2000). V Single-cell MALDI: A new tool for direct peptide profiling. Trends Biotechnol..

[201] Kay A.W., Strauss-Albee D.M., Blish C.A. (2016). Application of mass cytometry (CyTOF) for functional and phenotypic analysis of natural killer cells. Natural Killer Cells.

[202] Glynn M., King D., Ducrée J. (2016). Systems Biology in Single Cells. Essentials of Single-Cell Analysis.

[203] Smith K., Li Y., Piccinini F., Csucs G., Balazs C., Bevilacqua A., Horvath P. (2015). CIDRE: An illumination-correction method for optical microscopy. Nat. Methods.

[204] Liu D.C., Nocedal J. (1989). On the limited memory BFGS method for large scale optimization. Math. Program..

[205] Labhsetwar P., Cole J.A., Roberts E., Price N.D., Luthey-Schulten Z.A. (2013). Heterogeneity in protein expression induces metabolic variability in a modeled Escherichia coli population. Proc. Natl. Acad. Sci. USA.

[206] Balaban N.Q., Merrin J., Chait R., Kowalik L., Leibler S. (2004). Bacterial persistence as a phenotypic switch. Science.

[207] Di Talia S., Skotheim J.M., Bean J.M., Siggia E.D., Cross F.R. (2007). The effects of molecular noise and size control on variability in the budding yeast cell cycle. Nature.

[208] Van Heerden J.H., Wortel M.T., Bruggeman F.J., Heijnen J.J., Bollen Y.J.M., Planqué R., Hulshof J., O’Toole T.G., Wahl S.A., Teusink B. (2014). Lost in transition: Start-up of glycolysis yields subpopulations of nongrowing cells. Science.

[209] Sandler O., Mizrahi S.P., Weiss N., Agam O., Simon I., Balaban N.Q. (2015). Lineage correlations of single cell division time as a probe of cell-cycle dynamics. Nature.

[210] Godin M., Delgado F.F., Son S., Grover W.H., Bryan A.K., Tzur A., Jorgensen P., Payer K., Grossman A.D., Kirschner M.W. (2010). Using buoyant mass to measure the growth of single cells. Nat. Methods.

[211] Son S., Tzur A., Weng Y., Jorgensen P., Kim J., Kirschner M.W., Manalis S.R. (2012). Direct observation of mammalian cell growth and size regulation. Nat. Methods.

[212] Yang Q., Pando B.F., Dong G., Golden S.S., van Oudenaarden A. (2010). Circadian gating of the cell cycle revealed in single cyanobacterial cells. Science.

[213] Nagoshi E., Saini C., Bauer C., Laroche T., Naef F., Schibler U. (2004). Circadian gene expression in individual fibroblasts: Cell-autonomous and self-sustained oscillators pass time to daughter cells. Cell.

[214] Huang F., Sirinakis G., Allgeyer E.S., Schroeder L.K., Duim W.C., Kromann E.B., Phan T., Rivera-Molina F.E., Myers J.R., Irnov I. (2016). Ultra-high resolution 3D imaging of whole cells. Cell.

[215] Lantz A.W., Bao Y., Armstrong D.W. (2007). Single-cell detection: Test of microbial contamination using capillary electrophoresis. Anal. Chem..

[216] Bao N., Wang J., Lu C. (2008). Recent advances in electric analysis of cells in microfluidic systems. Anal. Bioanal. Chem..

[217] Legendre L.A., Morris C.J., Bienvenue J.M., Barron A., McClure R., Landers J.P. (2008). Toward a simplified microfluidic device for ultra-fast genetic analysis with sample-in/answer-out capability: Application to T-cell lymphoma diagnosis. JALA J. Assoc. Lab. Autom..

[218] Zheng X.T., Yang H.B., Li C.M. (2010). Optical detection of single cell lactate release for cancer metabolic analysis. Anal. Chem..

[219] Woods L.A., Powell P.R., Paxon T.L., Ewing A.G. (2005). Analysis of mammalian cell cytoplasm with electrophoresis in nanometer inner diameter capillaries. Electroanalysis.

[220] Omiatek D.M., Santillo M.F., Heien M.L., Ewing A.G. (2009). Hybrid capillary-microfluidic device for the separation, lysis, and electrochemical detection of vesicles. Anal. Chem..

[221] Wu R.-G., Yang C.-S., Cheing C.-C., Tseng F.-G. (2011). Nanocapillary electrophoretic electrochemical chip: Towards analysis of biochemicals released by single cells. Interface Focus.

[222] Majonis D., Herrera I., Ornatsky O., Schulze M., Lou X., Soleimani M., Nitz M., Winnik M.A. (2010). Synthesis of a functional metal-chelating polymer and steps toward quantitative mass cytometry bioassays. Anal. Chem..

[223] Thomas R. (2013). Practical guide to ICP-MS: A tutorial for beginners.

[224] Bendall S.C., Nolan G.P., Roederer M., Chattopadhyay P.K. (2012). A deep profiler’s guide to cytometry. Trends Immunol..

[225] Gerdtsson E., Pore M., Thiele J.-A., Gerdtsson A.S., Malihi P.D., Nevarez R., Kolatkar A., Velasco C.R., Wix S., Singh M. (2018). Multiplex protein detection on circulating tumor cells from liquid biopsies using imaging mass cytometry. Converg. Sci. Phys. Oncol..

[226] Marrinucci D., Bethel K., Kolatkar A., Luttgen M.S., Malchiodi M., Baehring F., Voigt K., Lazar D., Nieva J., Bazhenova L. (2012). Fluid biopsy in patients with metastatic prostate, pancreatic and breast cancers. Phys. Biol..

[227] Wang H.A.O., Grolimund D., Giesen C., Borca C.N., Shaw-Stewart J.R.H., Bodenmiller B., Gunther D. (2013). Fast chemical imaging at high spatial resolution by laser ablation inductively coupled plasma mass spectrometry. Anal. Chem..

[228] Behjati S., Tarpey P.S. (2013). What is next generation sequencing?. Arch. Dis. Child.-Educ. Pract..

[229] Gawad C., Koh W., Quake S.R. (2016). Single-cell genome sequencing: Current state of the science. Nat. Rev. Genet..

[230] Buenrostro J.D., Giresi P.G., Zaba L.C., Chang H.Y., Greenleaf W.J. (2013). Transposition of native chromatin for fast and sensitive epigenomic profiling of open chromatin, DNA-binding proteins and nucleosome position. Nat. Methods.

[231] Deng Q., Ramsköld D., Reinius B., Sandberg R. (2014). Single-cell RNA-seq reveals dynamic, random monoallelic gene expression in mammalian cells. Science.

[232] Osada T., Uehara H., Kim H., Ikai A. (2003). mRNA analysis of single living cells. J. Nanobiotechnol..

[233] Saha-Shah A., Weber A.E., Karty J.A., Ray S.J., Hieftje G.M., Baker L.A. (2015). Nanopipettes: Probes for local sample analysis. Chem. Sci..

[234] Pool R. (1988). Trapping with optical tweezers. Science.

[235] Ryttsen F., Farre C., Brennan C., Weber S.G., Nolkrantz K., Jardemark K., Chiu D.T., Orwar O. (2000). Characterization of single-cell electroporation by using patch-clamp and fluorescence microscopy. Biophys. J..

[236] Shojaei-Baghini E., Zheng Y., Sun Y. (2013). Automated micropipette aspiration of single cells. Ann. Biomed. Eng..

[237] Wong C.Y., Mills J.K. Cleavage-stage embryo rotation tracking and automated micropipette control: Towards automated single cell manipulation. Proceedings of the 2016 IEEE/RSJ International Conference on Intelligent Robots and Systems (IROS).

[238] Ungai-Salánki R., Gerecsei T., Fürjes P., Orgovan N., Sándor N., Holczer E., Horvath R., Szabó B. (2016). Automated single cell isolation from suspension with computer vision. Sci. Rep..

[239] Morishima K., Arai F., Fukuda T., Matsuura H., Yoshikawa K. (1998). Screening of single Escherichia coli in a microchannel system by electric field and laser tweezers. Anal. Chim. Acta.

[240] Schnelle T., Müller T., Hagedorn R., Voigt A., Fuhr G. (1999). Single micro electrode dielectrophoretic tweezers for manipulation of suspended cells and particles. Biochim. Biophys. Acta (BBA)-Gen. Subj..

[241] Schnelle T., Müller T., Reichle C., Fuhr G. (2000). Combined dielectrophoretic field cages and laser tweezers for electrorotation. Appl. Phys. B.

[242] Baumann C.G., Bloomfield V.A., Smith S.B., Bustamante C., Wang M.D., Block S.M. (2000). Stretching of single collapsed DNA molecules. Biophys. J..

[243] Leckband D. (2000). Measuring the forces that control protein interactions. Annu. Rev. Biophys. Biomol. Struct..

[244] Raucher D., Sheetz M.P. (2000). Cell spreading and lamellipodial extension rate is regulated by membrane tension. J. Cell Biol..

[245] Greulich K.O., Pilarczyk G., Hoffmann A., Meyer Zu Hörste G., Schäfer B., Uhl V., Monajembashi S. (2000). Micromanipulation by laser microbeam and optical tweezers: From plant cells to single molecules. J. Microsc..

[246] Kwon J.-S., Wereley S.T. (2013). Towards new methodologies for manipulation of colloidal particles in a miniaturized fluidic device: Optoelectrokinetic manipulation technique. J. Fluids Eng..

[247] Work A.H., Williams S.J. (2015). Characterization of 2D colloid aggregations created by optically induced electrohydrodynamics. Electrophoresis.

[248] Kumar A., Williams S.J., Wereley S.T. Rapid electrokinetic patterning of colloidal particles with optical landscapes. Proceedings of the 12th International Conference on Miniaturized Systems for Chemistry and Life Sciences (MicroTAS’08).

[249] Yuan G.-C., Cai L., Elowitz M., Enver T., Fan G., Guo G., Irizarry R., Kharchenko P., Kim J., Orkin S. (2017). Challenges and emerging directions in single-cell analysis. Genome Biol..

[250] Dincer C., Bruch R., Kling A., Dittrich P.S., Urban G.A. (2017). Multiplexed point-of-care testing—xPOCT. Trends Biotechnol..

[251] Tomizawa M., Shinozaki F., Motoyoshi Y., Sugiyama T., Yamamoto S., Sueishi M. (2013). Sonoporation: Gene transfer using ultrasound. World J. Methodol..

[252] Alsaggar M., Liu D. (2015). Physical methods for gene transfer. Advances in Genetics.

[253] Liu D., Wang L., Wang Z., Cuschieri A. (2012). Magnetoporation and magnetolysis of cancer cells via carbon nanotubes induced by rotating magnetic fields. Nano Lett..

[254] Chow Y.T., Chen S., Wang R., Liu C., Kong C., Li R.A., Cheng S.H., Sun D. (2016). Single cell transfection through precise microinjection with quantitatively controlled injection volumes. Sci. Rep..

[255] Zhang D., Lee H., Zhu Z., Minhas J.K., Jin Y. (2016). Enrichment of selective miRNAs in exosomes and delivery of exosomal miRNAs in vitro and in vivo. Am. J. Physiol. Cell. Mol. Physiol..

[256] Han X., Liu Z., Chan Jo M., Zhang K., Li Y., Zeng Z., Li N., Zu Y., Qin L. (2015). CRISPR-Cas9 delivery to hard-to-transfect cells via membrane deformation. Sci. Adv..

[257] Ding X., Stewart M.P., Sharei A., Weaver J.C., Langer R.S., Jensen K.F. (2017). High-throughput nuclear delivery and rapid expression of DNA via mechanical and electrical cell-membrane disruption. Nat. Biomed. Eng..

[258] Patel A.P., Tirosh I., Trombetta J.J., Shalek A.K., Gillespie S.M., Wakimoto H., Cahill D.P., Nahed B.V., Curry W.T., Martuza R.L. (2014). Single-cell RNA-seq highlights intratumoral heterogeneity in primary glioblastoma. Science.

[259] Janiszewska M., Liu L., Almendro V., Kuang Y., Paweletz C., Sakr R.A., Weigelt B., Hanker A.B., Chandarlapaty S., King T.A. (2015). In situ single-cell analysis identifies heterogeneity for PIK3CA mutation and HER2 amplification in HER2-positive breast cancer. Nat. Genet..

[260] Hou Y., Guo H., Cao C., Li X., Hu B., Zhu P., Wu X., Wen L., Tang F., Huang Y. (2016). Single-cell triple omics sequencing reveals genetic, epigenetic, and transcriptomic heterogeneity in hepatocellular carcinomas. Cell Res..

[261] Llorens-Bobadilla E., Zhao S., Baser A., Saiz-Castro G., Zwadlo K., Martin-Villalba A. (2015). Single-cell transcriptomics reveals a population of dormant neural stem cells that become activated upon brain injury. Cell Stem Cell.

